# ANT2 drives proinflammatory macrophage activation in obesity

**DOI:** 10.1172/jci.insight.147033

**Published:** 2021-10-22

**Authors:** Jae-Su Moon, Flavia Franco da Cunha, Jin Young Huh, Alexander Yu Andreyev, Jihyung Lee, Sushil K. Mahata, Felipe C.G. Reis, Chanond A. Nasamran, Yun Sok Lee

**Affiliations:** 1 Department of Medicine, Division of Endocrinology and Metabolism, University of California San Diego, La Jolla, California, USA.; 2 VA San Diego Healthcare System, San Diego, California, USA.; 3 Center for Computational Biology & Bioinformatics, Department of Medicine, University of California San Diego, La Jolla, California, USA.

**Keywords:** Endocrinology, Metabolism, Diabetes, Innate immunity

## Abstract

Macrophage proinflammatory activation is an important etiologic component of the development of insulin resistance and metabolic dysfunction in obesity. However, the underlying mechanisms are not clearly understood. Here, we demonstrate that a mitochondrial inner membrane protein, adenine nucleotide translocase 2 (ANT2), mediates proinflammatory activation of adipose tissue macrophages (ATMs) in obesity. *Ant2* expression was increased in ATMs of obese mice compared with lean mice. Myeloid-specific ANT2-knockout (ANT2-MKO) mice showed decreased adipose tissue inflammation and improved insulin sensitivity and glucose tolerance in HFD/obesity. At the molecular level, we found that ANT2 mediates free fatty acid–induced mitochondrial permeability transition, leading to increased mitochondrial reactive oxygen species production and damage. In turn, this increased HIF-1α expression and NF-κB activation, leading to proinflammatory macrophage activation. Our results provide a previously unknown mechanism for how obesity induces proinflammatory activation of macrophages with propagation of low-grade chronic inflammation (metaflammation).

## Graphical abstract

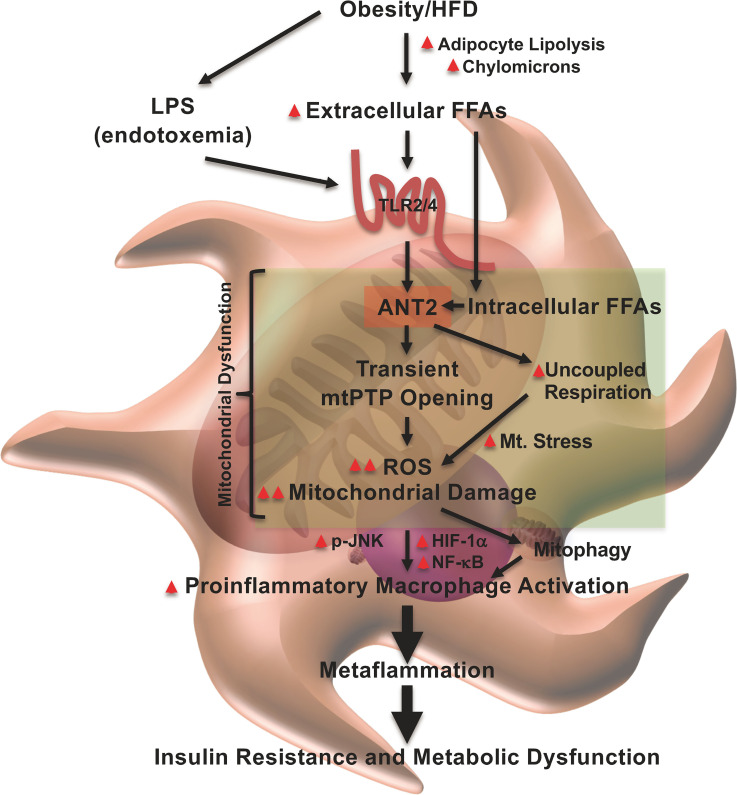

## Introduction

Obesity is characterized by low-grade chronic inflammation, often called metaflammation (1–5). Evidence indicates that macrophages play a central role in this process (6–8). Numerous studies showed that the depletion of macrophages or genetic manipulations to block proinflammatory macrophage activation improves insulin sensitivity and glucose tolerance in obese mice (3, 9–19). Macrophages are the major source of proinflammatory cytokines, eicosanoids, and other factors in adipose tissue that can cause insulin resistance and metabolic dysregulation in obesity (8, 20, 21). Moreover, adipose tissue macrophages (ATMs) in obese mice secrete nano-sized extracellular vesicles (exosomes) that can enter the systemic circulation and cause insulin resistance in liver and skeletal muscle through an endocrine-like mechanism (22).

During the development of obesity, the recruitment of blood monocytes into adipose tissue increases and these monocytes then differentiate into proinflammatory M1-like polarized macrophages (21, 23). Moreover, proliferation of ATMs also increases in obesity (24). Together, these changes lead to increased ATM accumulation in visceral adipose tissue (7, 8). In addition, obesity induces phenotypic switching of ATMs from M2-like polarized antiinflammatory to M1-like polarized proinflammatory activation states (21, 25). Although exactly how obesity induces proinflammatory ATM activation is not yet clearly understood, several lines of evidence indicate that increased free fatty acid (FFA) levels play a key role in this process by activating cell-surface Toll-like receptor 2 (TLR2) and TLR4 or altering lipid metabolism (9, 25–32).

In macrophages, the mitochondrion is not simply a subcellular compartment for energy production, but is key for the regulation of various macrophage activities (33). For example, M1-like macrophage polarization accompanies mitochondrial fragmentation, mitophagy, and decreased mitochondrial activity (34). Pharmacological inhibition of DRP1 or genetic deletion of *Drp1*, the key factor for the regulation of mitochondrial fission and mitophagy, blocks M1-like macrophage polarization (35, 36). Moreover, increased production of mitochondrial ROS (mtROS) or accumulation of a mitochondrial metabolite, succinate, drives M1-like macrophage polarization by stabilizing hypoxia-inducible factor 1α (HIF-1α) (37–39). Furthermore, mitochondrial damage induced during M1-like macrophage polarization restrains repolarization toward the M2-like phenotype (40). On the other hand, M2-polarized macrophages are active in oxidative phosphorylation and fatty acid oxidation, and this is necessary for macrophage-dependent tissue repair (41, 42). In obesity, ATMs show unique mitochondrial bioenergetics distinct from the lean state, along with increased inflammatory gene expression (25, 28, 43, 44).

Adenine nucleotide translocase (ANT) refers to a family of mitochondrial proteins that were first known as mediators of ADP/ATP exchange across the mitochondrial inner membrane through an antiport mechanism (45). Subsequent studies revealed that ANTs play a crucial role in 3 additional physiological processes: mitophagy (46), proton leak (47), and opening of the mitochondrial permeability transition pore (mtPTP) (48–50). Interestingly, the latter 2 effects are modulated by intracellular FFA levels (47, 50), suggesting that ANT2 could function as a mitochondrial FFA sensor. There are 4 isoforms of ANTs in humans and 3 in mice (ANT1, ANT2, and ANT4). ANT1 regulates basal mitochondrial respiration, whereas ANT2 mediates FFA-induced increases in uncoupled respiration in cells that are deficient in uncoupling protein 1 (UCP1) (51, 52). In obesity, increased intracellular FFAs stimulate ANT2, leading to increased oxygen (O_2_) consumption and a state of relative hypoxia. This triggers HIF-1α–dependent chemokine production, leading to adipose tissue inflammation, insulin resistance, and metabolic dysfunction (51, 53–57). Adipocyte-specific ANT2-KO mice are protected from obesity-induced adipose tissue hypoxia and show improved metaflammation, insulin sensitivity, and glucose tolerance (53). Moreover, hepatocyte-specific deletion of *Ant2* improves liver steatosis and insulin resistance in obese mice (58).

In the current study, we investigated the effect of ANT2 on macrophage development, growth, and proinflammatory activation. We found that ANT2 mediates FFA-induced mitochondrial permeability transition, triggering increased mtROS generation and mitochondrial damage. This leads to proinflammatory macrophage activation. Depletion of myeloid ANT2 inhibits adipose tissue inflammation and improves insulin sensitivity and glucose tolerance in obese mice.

## Results

### Generation of myeloid-specific ANT2-KO mice.

To study the effect of ANT2 in the regulation of macrophage function in lean and obese states, we generated myeloid lineage–specific ANT2 knockout (ANT2-MKO) mice by crossing *Ant2*-floxed (*Ant2^fl/fl^* female and *Ant2^fl/Y^* male) mice with heterozygous *Lysm-Cre* mice. Consistent with previous reports on the *Lysm*-*Cre*–driver mice (59), *Cre^+/–^* *Ant2^fl/Y^* (ANT2-MKO) mice showed an approximately 60% decrease in *Ant2* mRNA expression in monocytes and a greater than 95% decrease in bone marrow–derived macrophages (BMDMs) compared with *Cre^–/–^* *Ant2^fl/Y^* wild-type (WT) controls (Supplemental Figure 1, A–C; supplemental material available online with this article; https://doi.org/10.1172/jci.insight.147033DS1). Moreover, *Ant2* expression was decreased by 90%–95% in the stromal vascular fraction (SVC) and sorted ATMs from epididymal white adipose tissue (eWAT) of ANT2-MKO mice compared with WT littermate controls. *Ant2* expression was unchanged in adipocytes in ANT2-MKO mice, and *Ant1* expression was unchanged in isolated monocytes, BMDMs, and ATMs of ANT2-MKO mice (Supplemental Figure 1, D and E). *Ant2* mRNA expression was unchanged in the whole brain of ANT2-MKO mice compared with WT mice, although a minor *Ant2* gene deletion was detected in the brain (Supplemental Figure 1, F and G), consistent with a previous report (60). Body weight, eWAT mass, adipocyte size, liver mass, and histologic features of eWAT and liver were unchanged in ANT2-MKO mice compared with WT littermates on normal chow diet (NCD) (Supplemental Figure 1, H–M). Moreover, glucose tolerance and fasting plasma insulin levels were normal in NCD-fed ANT2-MKO mice (Supplemental Figure 1, N and O).

### Depletion of myeloid ANT2 improves insulin sensitivity and glucose tolerance in obesity.

**Figure 1:**
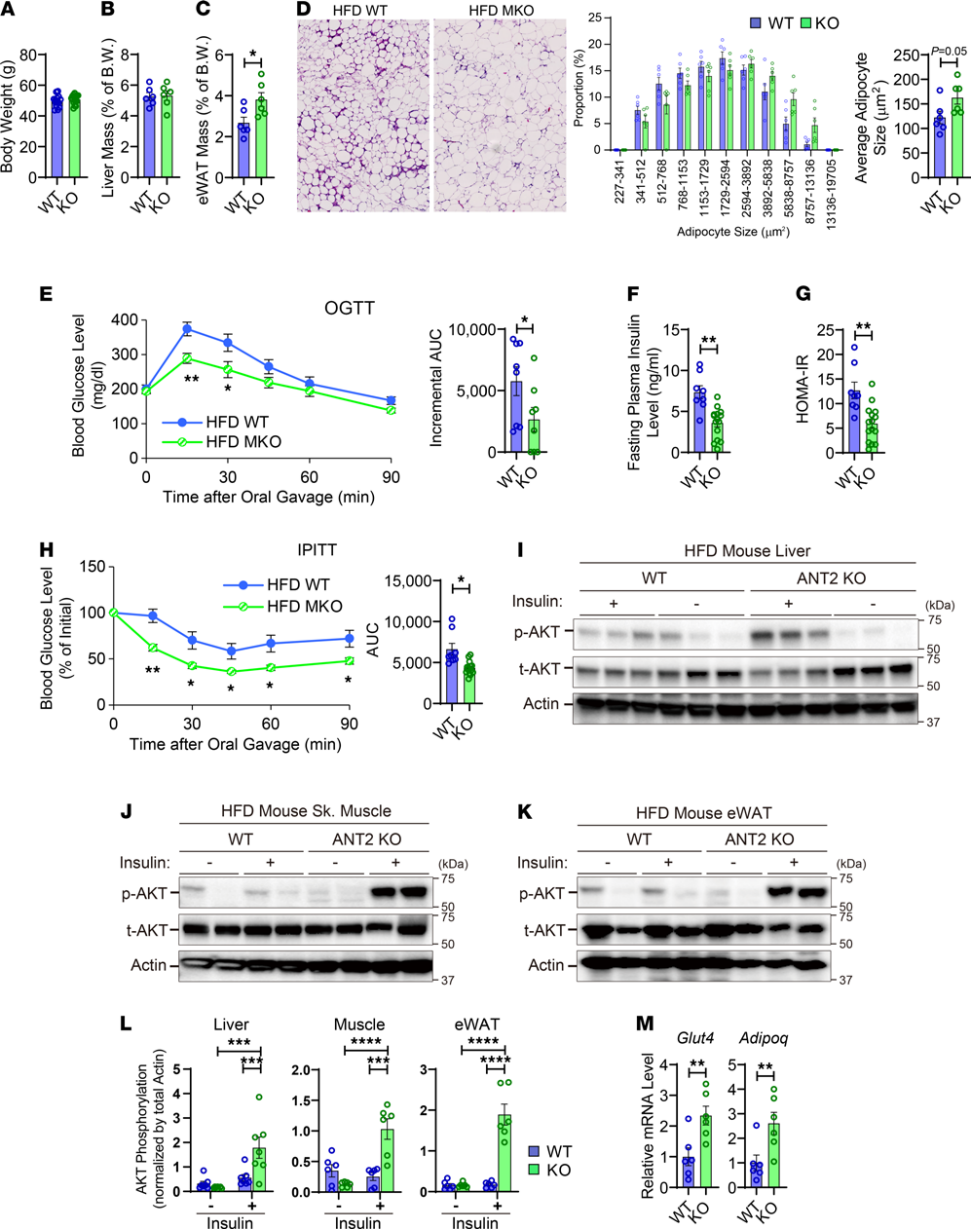
Myeloid-specific ANT2 depletion improves insulin sensitivity and glucose tolerance in obesity. (**A**) Body weight of HFD-fed WT and ANT2-MKO mice (*n* = 6 mice per group). (**B**) Liver mass of HFD-fed mice (*n* = 6 mice per group). (**C**) eWAT mass of HFD-fed mice (*n* = 6 mice per group). (**D**) Hematoxylin and eosin (H&E) staining (left) of eWAT sections of HFD-fed WT and ANT2-MKO mice (*n* = 6 mice per group). Microscopic images were taken under ×20 magnification. The distribution of adipocyte sizes (middle) and average adipocyte size (right) were plotted. (**E**) Oral glucose tolerance tests (OGTTs) in HFD-fed mice (*n* = 8 WT and 9 ANT2-MKO mice). (**F**) Fasting (6 hours) plasma insulin levels (*n* = 8 WT and 14 ANT2-MKO mice). (**G**) HOMA-IR in (**E**) mice. (**H**) Intraperitoneal insulin tolerance test (IPITT) in HFD-fed mice (*n* = 8 WT and 14 ANT2-MKO mice). (**I**) Insulin-stimulated Akt phosphorylation (p-AKT) in liver of HFD mice. t-AKT, total AKT. (**J**) Insulin-stimulated Akt phosphorylation in skeletal muscle (quadriceps) of HFD-fed mice. (**K**) Insulin-stimulated Akt phosphorylation in eWAT of HFD-fed mice. (**L**) Insulin-stimulated Akt phosphorylation in skeletal muscle, liver, and eWAT of HFD-fed WT and ANT2-MKO mice (*n* = 6 [skeletal muscle and eWAT] and 7 [liver]mice per group). (**M**) mRNA expression of *Glut4* and *Adipoq* in eWAT of HFD-fed mice (*n* = 6 WT and 6 ANT2-MKO mice). In all panels, values are mean ± SEM. *P < 0.05; **P < 0.01; ***P < 0.001; ****P < 0.0001. Statistical analysis was performed by 2-tailed, unpaired *t* test (**C** and **D**, right; **E**, right; **F** and **G**; **H**, right; and **M**) or 2-way ANOVA (**E**, left; **H**, left; and **L**) with Tukey’s multiple comparison test.

To investigate the effect of ANT2 on the development of obesity-induced glucose intolerance and insulin resistance, we fed ANT2-MKO and WT mice with 60% HFD and assessed their metabolic phenotypes. As seen in Figure 1, A and B, and Supplemental Figure 1P, body weight and liver mass were comparable in HFD-fed WT and ANT2-MKO mice. eWAT mass was slightly higher, with a tendency for greater adipocyte size in ANT2-MKO mice compared with HFD-fed WT mice (Figure 1, C and D). However, glucose tolerance was significantly improved in HFD-fed ANT2-MKO mice compared with HFD-fed WT mice (Figure 1E). These changes were accompanied by decreased fasting plasma insulin levels and homeostatic model assessment of insulin resistance (HOMA-IR) (Figure 1, F and G), suggesting that myeloid ANT2 depletion improved systemic insulin sensitivity. Consistent with this, insulin tolerance was improved in HFD-fed ANT2-MKO mice compared with HFD-fed WT mice (Figure 1H). To test whether these effects are associated with increased insulin signaling in classical insulin target tissues, basal and insulin-stimulated Akt phosphorylation was measured in liver, skeletal muscle, and eWAT of HFD-fed WT and ANT2-MKO mice. As seen in Figure 1, I–L, and Supplemental Figure 2, insulin-stimulated Akt phosphorylation was significantly higher in all 3 insulin target tissues of HFD-fed ANT2-MKO mice compared with HFD-fed WT controls. These changes were accompanied by greater expression of genes associated with insulin sensitivity such as *Glut4* and *Adipoq* (Figure 1M). Together, these results suggest that myeloid ANT2 depletion improves insulin sensitivity and glucose tolerance in obese mice.

### Myeloid ANT2 depletion improves metaflammation in obesity.

**Figure 2:**
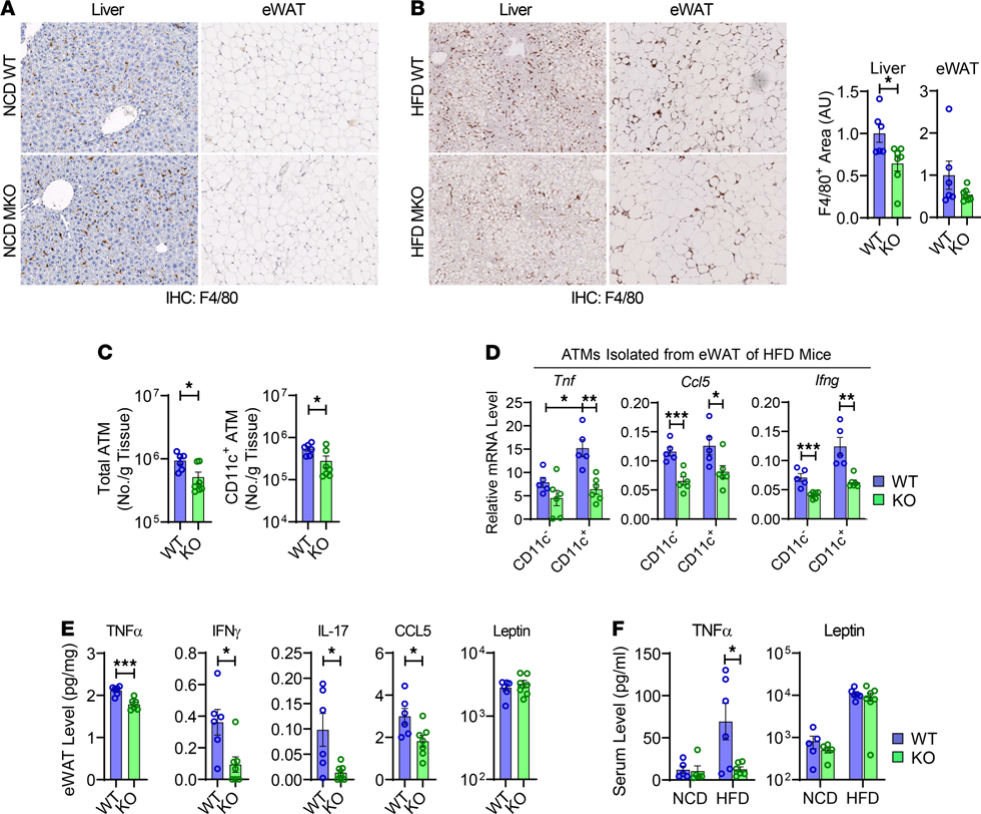
Myeloid-specific ANT2 depletion ameliorates adipose tissue inflammation in obesity. (**A**) IHC analysis of F4/80^+^ cells in liver and eWAT of NCD-fed WT and ANT2-MKO mice. Tissue samples harvested from 5 WT and 5 ANT2-MKO individual mice were analyzed and representative pictures are shown. (**B**) IHC analysis of F4/80^+^ cells in liver and eWAT of HFD-fed WT and ANT2-MKO mice (*n* = 6 and 7 mice). Representative pictures are shown on the left. Relative proportion of F4/80^+^ area per given section area was calculated and plotted (middle, liver; right, eWAT). AU, arbitrary unit. Microscopic images were taken under ×20 magnification (**A** and **B**). (**C**) Flow cytometry analysis of total and CD11c^+^ ATMs in eWAT of HFD-fed mice (*n* = 6 WT and 7 ANT2-MKO mice). (**D**) mRNA expression of inflammatory genes in ATMs isolated from eWAT of HFD-fed mice (*n* = 5 WT and 6 ANT2-MKO mice). (**E**) Proinflammatory cytokine levels in eWAT of HFD-fed WT and ANT2-MKO mice (*n* = 5, 6, 5, and 7 NCD- and HFD-fed WT and ANT2-MKO mice, respectively). (**F**) Serum TNF-α and leptin levels in WT and ANT2-MKO mice (*n* = 5, 6, 5, and 7 NCD- and HFD-fed WT and ANT2-MKO mice, respectively). All values are mean ± SEM. *P < 0.05; **P < 0.01; ***P < 0.001. Statistical analysis was performed by 2-tailed, unpaired *t* test (**B** and **C**) or 1-way ANOVA (**D** and **F**) with Tukey’s multiple comparison test.

To test whether the improvements in insulin resistance and glucose tolerance in ANT2-MKO mice are associated with decreased inflammation, we measured macrophage infiltration in adipose tissue and liver by immunohistochemical analysis. On NCD, F4/80^+^ macrophage infiltration in liver and eWAT was comparable in WT and ANT2-MKO mice (Figure 2A). Moreover, the proportion of different myeloid populations in the spleen and mesenteric lymph nodes was comparable in WT and ANT2-MKO mice and in vitro BMDM differentiation was comparable in hematopoietic stem cells isolated from WT and ANT-MKO mice, suggesting that myeloid development and macrophage differentiation were not affected by ANT2 depletion (Supplemental Figure 3, A–E). However, on HFD, the number of F4/80^+^ macrophages was significantly decreased in the liver and eWAT of ANT2-MKO mice compared with WT mice, as assessed by immunohistochemistry (Figure 2B). Moreover, flow cytometry analysis revealed that myeloid ANT2 depletion reduced the number of total and CD11c^+^ M1-like polarized ATMs in eWAT (Figure 2C and Supplemental Figure 3F). In addition, there was a trend of increased antiinflammatory regulatory T cells (Tregs) and decreased proinflammatory Th1 and Th17 cells in the eWAT of HFD-fed ANT2-MKO mice compared with HFD-fed WT mice (Supplemental Figure 3, G and H).

Furthermore, mRNA expression of the proinflammatory genes *Tnf*, *Ifng*, and *Ccl5* was lower in ATMs isolated from HFD-fed ANT2-KO mice compared with HFD WT mice (Figure 2D). Interestingly, while the reduction in proinflammatory gene expression by ANT2 depletion was seen in both CD11c^+^ and CD11c^–^ ATMs, it was more pronounced in CD11c^+^ ATMs, the majority of which are M1-like polarized and derived from blood monocytes (21). Moreover, unbiased RNA sequencing (RNA-seq) analysis revealed that the expression of genes implicated in inflammatory responses was reduced in CD11c^+^ ATMs isolated from HFD-fed ANT2-MKO mice compared with HFD-fed WT mice, whereas the expression of genes implicated in oxidative phosphorylation and fatty acid metabolism was greater (Supplemental Table 1). These results suggest that ANT2 depletion inhibited obesity-induced proinflammatory ATM activation. Consistent with these results, the levels of proinflammatory cytokines and chemokines, including TNF-α, IFN-γ, IL-17, and CCL5, were lower in eWAT of HFD-fed ANT2-MKO mice compared with HFD-fed WT mice (Figure 2E). Moreover, serum TNF-α levels were also lower in HFD-fed ANT2-MKO mice, while leptin levels were unchanged (Figure 2F).

Since the effect of ANT2 MKO was more robust in CD11c^+^ ATMs, we tested whether *Ant2* is enriched in this ATM subset. To this end, we analyzed single-cell RNA-seq (scRNA-seq) data reported previously (61). Consistent with previous reports, obesity enhanced the proportion of *Itgax^+^* (encoding CD11c) and *Cd9^+^* ATMs (cluster 0) and *Plac8^+^* monocytes (cluster 4) and reduced the proportion of *Lyve1^+^* and *Cd163^+^* ATMs (cluster 1) and *Lyz1^+^* monocytes (Supplemental Figure 3, I–K) (61–63). Interestingly, *Ant2* expression was enriched in the cluster 0 ATMs expressing *Cd9* and *Itgax,* although *Ant2* was expressed broadly in all ATM clusters (Supplemental Figure 3, L and M). Consistent with the notion that the majority of *Cd9^+^* and *Itgax^+^* ATMs are derived from monocytes, *Ant2* was also abundantly expressed in adipose tissue monocytes (Supplemental Figure 3N). Consistent with this, *Ant2* was abundantly expressed in isolated bone marrow–derived monocytes and in vitro–differentiated BMDMs (Supplemental Figure 1A and Supplemental Figure 3O). Moreover, obesity increased *Ant2* expression in *Itgax^–^, Lyve1*^+^, and *Cd163*^+^ ATMs (Supplemental Figure 3M), although *Ant2* expression was unchanged by LPS treatment in BMDMs (Supplemental Figure 3O). These results suggest that obesity can increase ATM *Ant2* expression not only by increasing the proportion of *Ant2*-high-expressing ATM subsets (*Cd9*^+^ and *Itgax*^+^ ATMs) but also by increasing *Ant2* expression in *Ant2*-low-expressing ATMs (*Itgax^–^*, *Lyve1*^+^, and *Cd163*^+^ ATMs). Consistent with this, we found that mRNA expression of *Ant2* was increased in HFD/obese ATMs compared with lean ATMs as assessed by scRNA-seq data analysis (Supplemental Figure 3N), as well as by real-time reverse transcription PCR (RT-PCR) analysis in sorted ATMs from NCD- and HFD-fed mice (Supplemental Figure 3P). Together, these results suggest that *Ant2* expression is enriched in bone marrow–derived proinflammatory ATMs, and myeloid ANT2 depletion improves metaflammation without affecting myeloid development and macrophage differentiation.

### ANT2 is essential for M1-like macrophage polarization.

**Figure 3:**
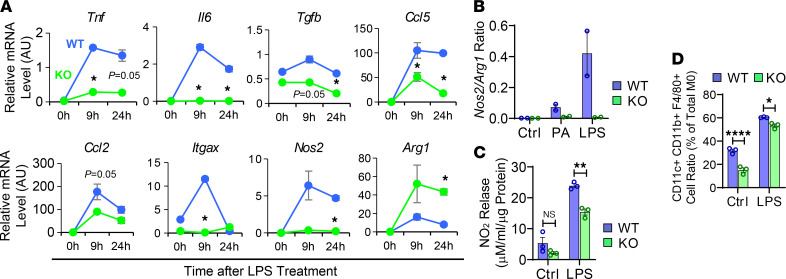
ANT2 is necessary for the proinflammatory activation of macrophages. (**A**) mRNA expression of inflammatory genes in WT and ANT2-MKO BMDMs before and 9 and 24 hours after LPS treatment (*n* = 2 wells/group). (**B**) The *Nos2*/*Arg1* ratio in WT and ANT2-MKO BMDMs treated with or without PA or LPS for 24 hours (*n* = 2 wells/group). (**C**) Nitrite levels in WT and ANT2-MKO BMDM culture media, treated with or without LPS for 24 hours (*n* = 3 wells/group). (**D**) Flow cytometry analysis of the proportion of CD11c^+^ cells among WT and ANT2-MKO BMDMs treated with or without LPS for 24 hours (*n* = 3 wells/group). All values are mean ± SEM. *P < 0.05; **P < 0.01; ****P < 0.0001. NS, not significant. Statistical analysis was performed by 2-way (**A**) or 1-way (**B**–**D**) ANOVA with Tukey’s multiple comparison test.

Next, we assessed whether ANT2 is necessary for proinflammatory macrophage activation. Since *Ant2* was abundantly expressed in bone marrow–derived ATMs and the effect of ANT2 depletion was more robust in this ATM population, we performed mechanistic studies in BMDMs. Interestingly, LPS-induced proinflammatory gene expression was substantially attenuated in ANT2-MKO BMDMs compared with WT BMDMs (Figure 3A). One of the key features of M1-like polarized macrophages is the increased expression of iNOS and production of nitric oxide (NO), which is functionally suppressed by arginase that is abundantly expressed in M2-like polarized macrophages (64). As seen in Figure 3, B and C, ANT2 depletion substantially reduced the *Nos2* (encoding iNOS)/*Arg1* ratio and the release of nitrite in macrophages. Moreover, flow cytometry analyses revealed that ANT2 depletion reduces the increase in the proportion of CD11c^+^ BMDMs stimulated by LPS treatment (Figure 3D and Supplemental Figure 4A). These results suggest that ANT2 depletion attenuates LPS-induced M1-like macrophage polarization.

### ANT2 is essential for metabolic activation of macrophages.

**Figure 4:**
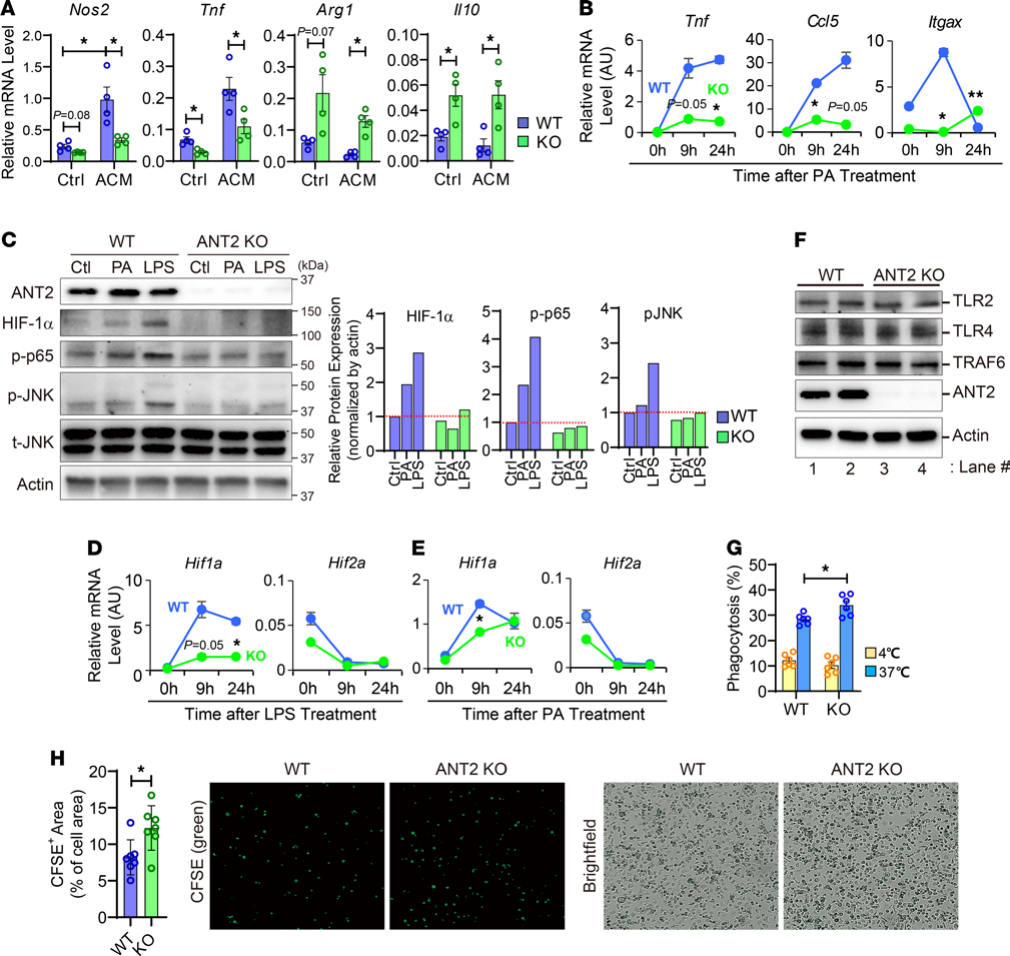
ANT2 is necessary for the metabolic activation of macrophages. (**A**) mRNA expression of proinflammatory genes in BMDMs treated with or without high glucose–primed 3T3-L1 adipocyte–conditioned media (ACM) for 24 hours (*n* = 4 wells/group). (**B**) mRNA expression of inflammatory genes in WT and ANT2-MKO BMDMs before and 9 and 24 hours after PA treatment (*n* = 2 wells/group). (**C**) Western blot analysis of inflammatory pathways in WT and ANT2-MKO BMDMs treated with or without PA or LPS for 30 minutes. Relative band intensity is plotted on the right. (**D**) mRNA expression of *Hif1a* and *Hif2a* in WT and ANT2-MKO BMDMs before and 9 and 24 hours after LPS treatment (*n* = 2 wells/group). (**E**) mRNA expression of *Hif1a* and *Hif2a* in WT and ANT2-MKO BMDMs before and 9 and 24 hours after PA treatment (*n* = 2 wells/group). (**F**) Western blot analysis of TLRs in WT and ANT2-MKO BMDMs (*n* = 2 wells/group). (**G**) Phagocytosis assays in WT and ANT2-MKO BMDMs (*n* = 6 wells/group). (**H**) Efferocytosis assays in WT and ANT2-MKO BMDMs (*n* = 7 wells/group). Microscopic images were taken under ×20 magnification. All values are mean ± SEM. *P < 0.05; **P < 0.01. Statistical analysis was performed by 2-tailed, unpaired *t* test (**H**) or 1-way (**A** and **G**) or 2-way (**B** and **D**) ANOVA with Tukey’s multiple comparison test.

Adipocytes secrete soluble factors that can modulate macrophage activation states, such as FFAs and IL-13 (65, 66), and obesity induces increased FFA release in adipocytes, stimulating M1-like ATM polarization (26, 66–68). To assess whether macrophage ANT2 plays a role in the mediation of proinflammatory activation of ATMs by factors released from adipocytes, we incubated WT and ANT2-MKO BMDMs with differentiated 3T3-L1 adipocyte–conditioned media (ACM). To induce proinflammatory programs in adipocytes, we preincubated adipocytes in high-glucose media before harvesting ACM (69). As seen in Figure 4A, ACM-induced expression of M1-like polarized macrophage marker genes such as *Tnf* and *Nos2* was attenuated in ANT2-MKO BMDMs, whereas the expression of M2 macrophage marker genes such as *Arg1* and *Il10* was increased in ANT2-MKO BMDMs compared with WT BMDMs. Consistent with these results, palmitic acid–induced (PA-induced) expression of *Tnf*, *Ccl5*, and *Itgax* was attenuated in ANT2-MKO BMDMs (Figure 4B). Moreover, the PA-induced increase in the *Nos2*/*Arg1* ratio was decreased in ANT2-MKO BMDMs compared with WT BMDMs (Figure 3B). PA- or LPS-stimulated HIF-1α expression and phosphorylated/activated p65 NF-κB and JNK levels were markedly attenuated in ANT2-MKO BMDMs compared with WT BMDMs (Figure 4, C–E). These results indicate that ANT2 is necessary for proinflammatory ATM activation induced by soluble factors released from obese adipocytes, including FFAs.

To assess whether the decrease in proinflammatory gene expression in ANT2-MKO BMDMs is associated with changes in TLR expression, we measure the expression of *Tlr1*, -*2*, and -*4*. As seen in Figure 4F and Supplemental Figure 4, B and C, mRNA expression of *Tlr1* and mRNA and protein expression of *Tlr4* were not changed by ANT2 depletion. Although *Tlr2* mRNA expression was slightly decreased (Supplemental Figure 4C), TLR2 protein expression was comparable in ANT2-MKO and WT BMDMs (Figure 4F). Interestingly, LPS-induced TRAF6 protein expression and mitochondrial translocation were decreased by ANT2 MKO (Supplemental Figure 4, D and E), consistent with decreased JNK and NF-κB activation.

Obesity is associated with increased adipocyte apoptosis (as featured histologically by crown-like structures) (70), and inefficient clearance of dead cell debris by macrophages can lead to chronic inflammation. To test whether ANT2 depletion affects phagocytic activity of macrophages, we incubated WT and ANT2-MKO BMDMs with FITC-conjugated latex beads and measured the amount of bead uptake. As seen in Figure 4G, ANT2-MKO BMDMs showed higher levels of FITC-labeled bead uptake without changes in nonspecific/passive bead-cell contact (which is not affected by temperature), suggesting that ANT2 deficiency enhanced phagocytic activity in macrophages. Moreover, ANT2-MKO BMDMs displayed increased uptake of apoptotic cell bodies compared with WT BMDMs, suggesting that ANT2 deficiency enhanced efferocytosis in macrophages (Figure 4H).

### ANT2 depletion decreases circulating monocyte migration into adipose tissue.

**Figure 5:**
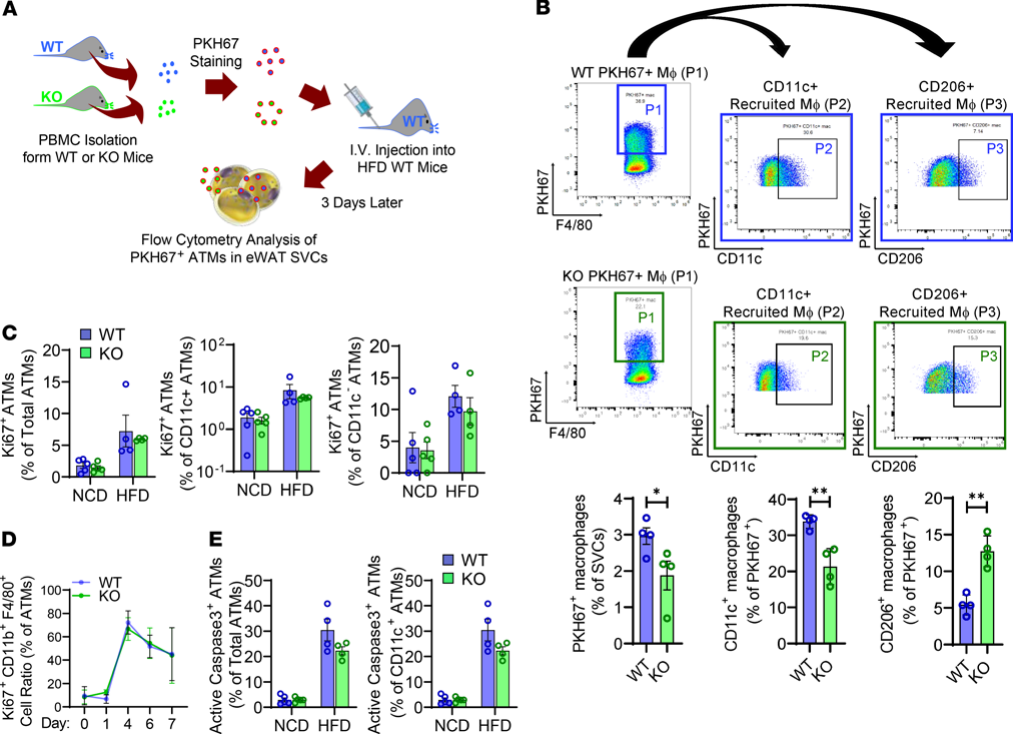
Myeloid-specific ANT2 is necessary for the recruitment of blood monocytes into adipose tissue in obesity without affecting macrophage proliferation. (**A**) Schematic representation of monocyte tracking experiments in **B**. (**B**) Flow cytometry analysis of PKH67^+^ ATMs derived from injected WT or ANT2-MKO peripheral blood mononuclear cells (PBMCs) (*n* = 4 mice per group). PKH67^+^F4/80^+^ ATMs (P1) were selected for further analysis of CD11c (P2) and CD206 expression (P3). *P < 0.05, **P < 0.01. Statistical analysis was performed by 2-tailed, unpaired *t* test. (**C**) Flow cytometry analysis of Ki67^+^ proliferating ATMs in NCD- and HFD-fed WT and ANT2-MKO mice (*n* = 5, 5, 4, and 4 NCD- and HFD-fed WT and ANT2-MKO mice, respectively). (**D**) Flow cytometry analysis of Ki67^+^ monocytes/macrophages during in vitro differentiation of BMDMs (*n* = 3 wells/group). (**E**) Flow cytometry analysis of active caspase-3^+^ apoptotic ATMs in NCD- and HFD-fed WT and ANT2-MKO mice (*n* = 5, 4, 5, and 4 NCD- and HFD-fed WT and ANT2-MKO mice, respectively). In all panels, all values are mean ± SEM.

Obesity-induced ATM accumulation is associated with increased blood monocyte recruitment and ATM proliferation (24, 32). Since ANT2-MKO mice showed decreased ATM accumulation in obesity, we performed monocyte-tracking experiments to test whether this change is associated with decreased recruitment of circulating monocytes into adipose tissue. As illustrated in Figure 5A, peripheral blood mononuclear cells (PBMCs) were isolated from WT and ANT2-MKO mice, stained with PKH67 fluorescent dye, and intravenously injected into HFD-fed/obese WT mice. Three days after injection, the number of PKH67^+^ ATMs was analyzed in eWAT of recipient mice using flow cytometry. As seen in Figure 5B and Supplemental Figure 5A, the number of PKH67^+^ ATMs was lower in mice injected with ANT2-MKO monocytes compared with mice treated with WT monocytes. This was associated with a slight decrease in mRNA expression of *Ccr2* in ANT2-MKO monocytes compared with WT monocytes (Supplemental Figure 5B). To test whether ANT2 MKO decreased ATM proliferation, we performed flow cytometry analysis of Ki67^+^ proliferating ATMs in NCD- and HFD-fed WT and ANT2-MKO mice. As seen in Figure 5C and Supplemental Figure 5C, the proportion of Ki67^+^ proliferating ATMs was unchanged in ANT2-MKO mice compared with WT mice in both CD11c^+^ and CD11c^–^ populations. Moreover, macrophage colony–stimulating factor–induced (M-CSF–induced) proliferation of ANT2-MKO bone marrow–derived monocytes was comparable to that of WT monocytes during BMDM differentiation (Figure 5D). To assess whether ANT2 deficiency affected ATM apoptosis, we also measured the proportion of apoptotic ATMs after staining with anti–active/cleaved caspase-3 antibodies. As seen in Figure 5E and Supplemental Figure 5C, the proportion of apoptotic ATMs was not increased in HFD-fed ANT2-MKO mice compared with HFD-fed WT mice. Instead, there was a trend of decreased ATM apoptosis in HFD-fed ANT2-MKO mice, although it did not reach statistical significance. Together, these results suggest that ANT2 depletion blocks obesity-induced ATM accumulation by attenuating monocyte recruitment into adipose tissue without decreasing ATM proliferation or increasing ATM death.

### ANT2 depletion preserves mitochondrial capacity during proinflammatory macrophage activation.

**Figure 6:**
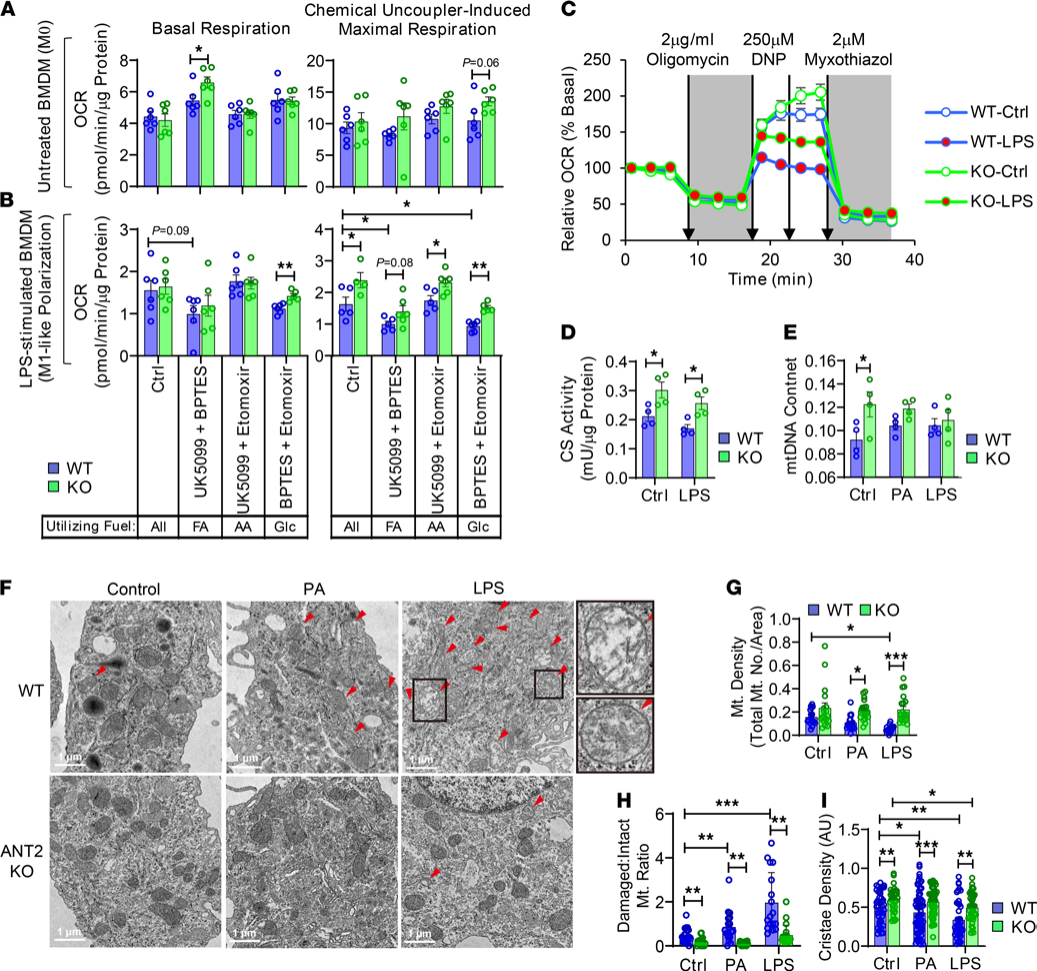
ANT2 depletion preserves mitochondrial respiratory capacity during proinflammatory macrophage activation. (**A** and **B**) Basal and chemical uncoupler–induced maximal OCRs were measured in the presence or absence of inhibitors of glucose (Glc) (UK5099), FA (etomoxir), and/or amino acid (AA) (BPTES) transport into mitochondria in WT and ANT2-MKO BMDMs treated without (**A**) or with (**B**) LPS for 24 hours (*n* = 6 wells/group). MΦ, macrophage. (**C**) Relative OCR in WT and ANT2-MKO BMDMs treated with or without LPS for 24 hours (*n* = 10 wells/group). DNP, 2,4-dinitrophenol. (**D**) CS activity in WT and ANT2-MKO BMDMs treated with or without LPS for 24 hours (*n* = 4 wells/group). (**E**) mtDNA content in WT and ANT2-MKO BMDMs treated with or without PA or LPS for 24 hours (*n* = 4 wells/group). (**F**–**I**) Transmission electron microscopy analysis of WT and ANT2-MKO BMDMs treated with or without PA or LPS for 24 hours. Representative pictures are shown in **F** (damaged mitochondria in the boxed areas are shown in higher magnitude on the right). The number of damaged and healthy-looking mitochondria was counted in a given cellular area and used to calculate mitochondrial density (**G**) and the ratio between damaged and intact mitochondria (**H**). Cristae density was measured in intact mitochondria in WT and ANT2-MKO BMDMs (**I**) (*n* = 1–2 mitochondria in 18–20 cells/group). In all panels, all values are mean ± SEM. *P < 0.05; **P < 0.01; ***P < 0.001. Statistical analysis was performed by 2-way ANOVA with Tukey’s multiple comparison test.

To assess the effect of ANT2 on macrophage mitochondrial activity, we measured the mitochondria-dependent O_2_ (oxygen) consumption rate (OCR) in WT and ANT2-MKO BMDMs treated with or without LPS for 24 hours. In the untreated, naive M0 state, WT and ANT2-MKO BMDMs showed comparable levels of basal and chemical uncoupler–induced maximal OCR, and the basal OCR was approximately 40% of the maximal capacity in both genotypes (Figure 6, A and B). Upon LPS treatment, both basal and maximal OCR were decreased in WT BMDMs (Figure 6, A and B). However, the reduction in the maximal OCR was greater, resulting in a lower basal mitochondrial operating rate — from approximately 40% in untreated M0 BMDMs to approximately 90% in LPS-treated M1-like polarized BMDMs (Figure 6C). Interestingly, in ANT2-MKO BMDMs, the LPS-induced decrease in mitochondrial respiratory capacity was markedly attenuated, while the basal OCR was unchanged. This resulted in decreased basal mitochondrial utilization, down to approximately 77% of the maximal capacity. To assess whether these changes in ANT2-MKO BMDMs are associated with changes in the mitochondrial capacity to utilize specific mitochondrial fuels, we measured OCR in WT and ANT2-MKO BMDMs in the presence or absence of UK0599, etomoxir, and/or BPTES, which specifically block pyruvate, fatty acid, or amino acid transport into mitochondria, respectively. In WT BMDMs, basal and maximal mitochondrial respiration was barely affected by combinatorial treatments with UK0599, etomoxir, and/or BPTES in the M0 state (Figure 6, A and B), suggesting mitochondrial fuel flexibility. LPS treatment decreased mitochondrial capacity to utilize glucose (pyruvate) and fatty acids in WT BMDMs, which was attenuated in ANT2-MKO BMDMs, suggesting that ANT2 depletion preserved mitochondrial fuel flexibility. Interestingly, although ANT2 depletion enhanced mitochondrial capacity to utilize fatty acids, it did not reduced basal PA-induced mitochondrial respiration, but actually lowered PA-mediated uncoupled respiration (Supplemental Figure 6, A and B).

To test whether the increase in mitochondrial capacity is associated with increased intact mitochondrial mass, we measured citrate synthase (CS) activity. As seen in Figure 6D, CS activity was increased by ANT2 depletion in both the M0 and M1-like polarized states. There was a trend toward greater mitochondrial DNA (mtDNA) content in ANT2-MKO BMDMs in the M0 state; however, in the M1-like polarization state, mtDNA content was comparable in WT and ANT2-MKO BMDMs (Figure 6E). Consistent with this, PGC-1α expression was comparable in WT and ANT2-MKO BMDMs (Supplemental Figure 6C), suggesting that mitochondrial biogenesis was unchanged in ANT2-MKO BMDMs. Electron microscopic analysis revealed that LPS treatment decreased mitochondrial density in WT BMDMs, which was rescued in ANT2-MKO BMDMs (Figure 6, F and G). Interestingly, the LPS-induced decrease in intact mitochondria mass and density in WT BMDMs was associated with an increase in the ratio of damaged to intact mitochondria (Figure 6H). Moreover, cristae density within intact mitochondria was decreased by LPS treatment in WT BMDMs. In ANT2-MKO BMDMs, the LPS-induced decreases in mitochondrial density and cristae density per intact mitochondria were substantially attenuated compared with WT BMDMs (Figure 6, H and I), suggesting that ANT2 depletion preserves mitochondrial health during M1-like macrophage polarization. Similarly, ANT2 depletion protected against the PA-induced increase in the damaged mitochondria/intact mitochondria ratio and decreased mitochondrial cristae density without affecting mtDNA content (Figure 6, E–I).

### ANT2 depletion blocks mitophagy during M1-like macrophage polarization.

Mitophagy is the key cellular process for mitochondrial quality control by removing damaged mitochondria (71). Mitochondria-targeted Keima-Red (mKeima-Red) is a recombinant fluorescent protein that shows color switching from green in neutral pH to red in acidic pH, like in autolysosomes (72). This feature of mKeima-Red allows quantitative measurement of the changes in the number of cytosolic versus autolysosomal mitochondria. To assess whether ANT2 depletion decreased the number of damaged mitochondria through increasing mitophagy, we adopted an mKeima-Red reporter system and measured changes in mitophagy. As seen in Supplemental Figure 6D, LPS treatment increased the red/green fluorescence intensity ratio in WT BMDMs, indicating that M1-like macrophage polarization induces mitophagy (36). In ANT2-MKO BMDMs, the red/green ratio was significantly decreased compared with WT BMDMs, suggesting that ANT2 depletion reduced mitophagy in M1-like macrophage polarization. Electron microscopic analysis revealed that ANT2 depletion suppressed PA- or LPS-stimulated autophagosome formation (Supplemental Figure 6E).

### ANT2 mediates increased mtROS generation by inducing opening of the mtPTP.

**Figure 7:**
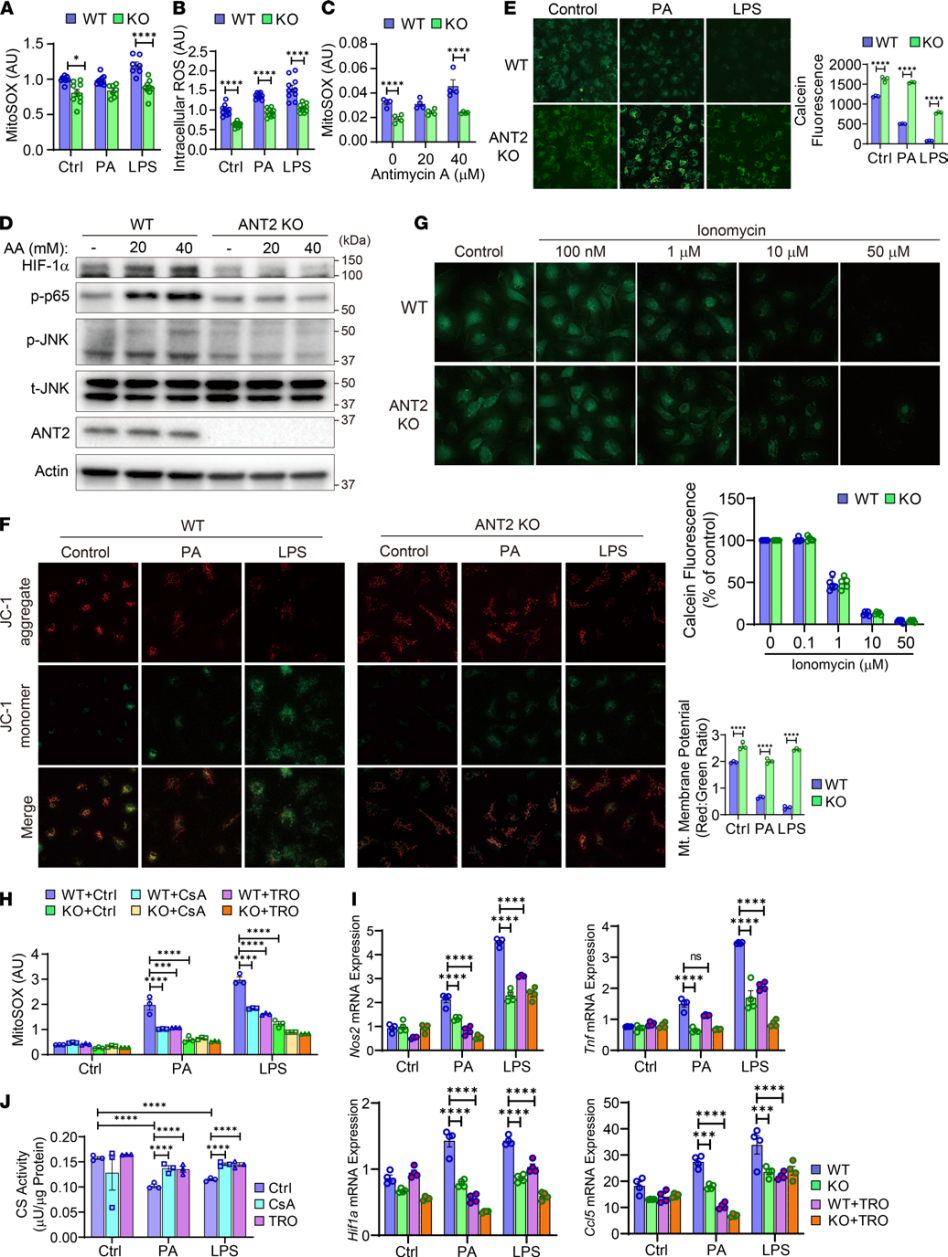
ANT2 mediates opening of the mtPTP and increased mtROS generation during M1-like macrophage polarization. (**A**) mtROS levels in WT and ANT2-MKO BMDMs treated with or without LPS for 30 minutes (*n* = 8–10 wells/group). (**B**) Cytosolic ROS levels in WT and ANT2-MKO BMDMs treated with or without LPS for 30 minutes (*n* = 10 wells/group). (**C**) mtROS levels in WT and ANT2-MKO BMDMs treated with or without antimycin A for 30 minutes (*n* = 4 wells/group). (**D**) Western blot analysis of inflammatory signaling pathway in WT and ANT2-MKO BMDMs treated with or without antimycin A (AA) for 30 minutes. (**E**) Opening of the mtPTP. WT and ANT2-MKO BMDMs were treated with LPS or PA for 10 minutes and opening of the mtPTP was measured by the calcein-cobalt method. Reduction in calcein fluorescence indicates opening of the mtPTP (*n* = 3 wells/group). (**F**) Mitochondrial membrane potential in WT and ANT2-MKO BMDMs treated with LPS or PA for 10 minutes (*n* = 3 wells/group). (**G**) Opening of the mtPTP in WT and ANT2-MKO BMDMs treated with ionomycin for 5 minutes (*n* = 5 wells/group). (**H**) mtROS levels in WT and ANT2-MKO BMDMs treated with or without PA (100 μM) or LPS (600 ng/mL) for 30 minutes in the presence or absence of CsA or TRO19622 (*n* = 3 wells/group). (**I**) mRNA expression of proinflammatory genes in WT and ANT2-MKO BMDMs treated with or without PA (100 μM) or LPS (600 ng/mL) in the presence or absence of TRO19622 (TRO) (*n* = 4 wells/group). (**J**) CS activity in WT BMDMs treated with or without PA (100 μM) or LPS (600 ng/mL) for 6 hours in the presence or absence of CsA or TRO19622 (*n* = 3 wells/group). Microscopic images in **E**–**G** were taken under ×20 magnification. In all panels, all values are mean ± SEM. *P < 0.05; **P < 0.01; ***P < 0.001; ****P < 0.0001. Statistical analysis was performed by 2-way ANOVA with Tukey’s multiple comparison test.

Since ANT2 depletion improved mitochondrial health without enhancing mitophagy or mitochondrial biogenesis, we hypothesized that ANT2 mediates higher mtROS generation upon FFA or LPS treatment, causing mitochondrial damage. To address this hypothesis, mtROS levels were measured using the mitochondria-specific fluorescent ROS indicator, MitoSOX. As seen in Figure 7, A and B, PA or LPS treatment induced mtROS, as well as cytosolic ROS, levels within 30 minutes, and this was markedly reduced in ANT2-MKO BMDMs. Antimycin A induces mitochondrial stress and mtROS by inhibiting mitochondrial complex III (73). To test whether ANT2 depletion can alleviate mitochondrial stress/damage, we measured mtROS levels after antimycin A treatment in WT and ANT2-MKO BMDMs. As seen in Figure 7C, antimycin A–induced mtROS levels were reduced in ANT2-MKO BMDMs. Moreover, antimycin A–stimulated proinflammatory pathways, including increased HIF-1α expression and phosphorylated/activated p65 NF-κB and JNK levels, were reduced in ANT2-MKO BMDMs (Figure 7D and Supplemental Figure 7).

The mtPTP is a multi-protein complex forming a nonspecific channel spanning the mitochondrial inner membrane (74). ANT2 is an important modulator of the opening of the mtPTP (48, 49), which causes mitochondrial swelling and decreased mitochondrial membrane potential, leading to increased mtROS production and mitochondrial damage. Therefore, we assessed whether ANT2-dependent mtROS generation and mitochondrial damage during M1-like macrophage polarization are associated with opening of the mtPTP. In WT BMDMs, PA or LPS treatment induced opening of the mtPTP and mitochondrial membrane depolarization in 10 minutes (Figure 7, E and F). Interestingly, in ANT2-MKO BMDMs, these effects of PA or LPS were substantially attenuated (Figure 7, E and F), whereas opening of the mtPTP induced by treatment with a calcium ionophore (ionomycin) was not affected by ANT2 MKO (Figure 7G). Moreover, treatment with mtPTP blockers such as cyclosporine A (CsA) or TRO19622 reduced PA- or LPS-induced mtROS production, similar to the effect of ANT2 MKO (Figure 7H). In addition, LPS-induced proinflammatory gene expression was substantially attenuated by TRO19622 treatment (Figure 7I). Furthermore, the PA- or LPS-induced reduction in CS activity was rescued by CsA or TRO19622 treatment in WT BMDMs, similar to the effect of ANT2 MKO (Figure 7J). These results suggest that ANT2-dependent opening of the mtPTP is essential for PA- or LPS-induced mtROS generation, mitochondrial damage, and proinflammatory gene expression in macrophages.

### mtROS plays a key role in ANT2-dependent proinflammatory macrophage activation.

**Figure 8:**
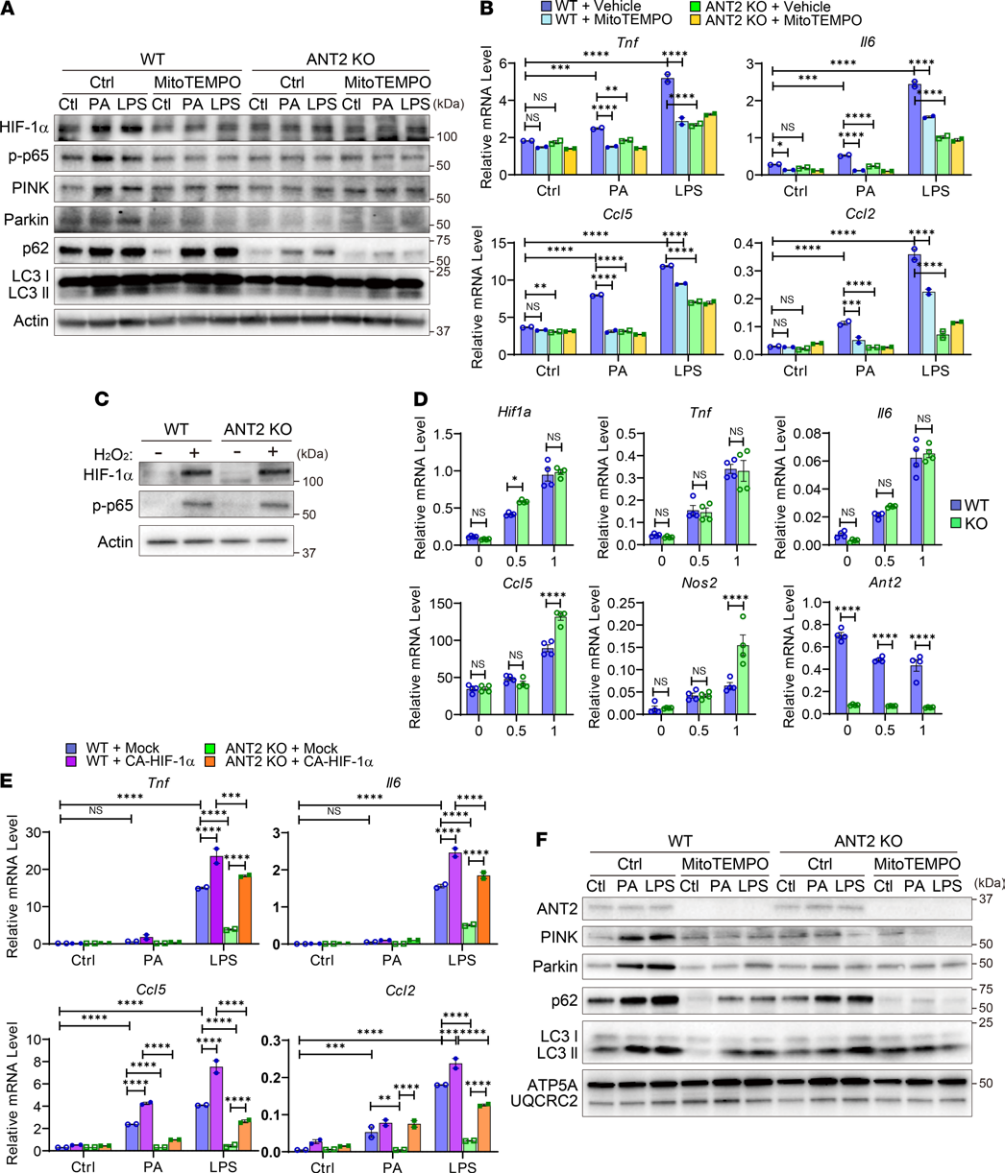
Increased ROS and subsequent HIF-1α stabilization mediates ANT2-dependent macrophage proinflammatory activation. (**A**) Western blot analysis of inflammatory and mitophagic pathways in WT and ANT2-MKO BMDMs treated with or without PA or LPS in the presence or absence of MitoTEMPO for 6 hours. (**B**) mRNA expression of inflammatory genes in WT and ANT2-MKO BMDMs treated with or without PA or LPS in the presence or absence of MitoTEMPO for 6 hours (*n* = 2 wells/group). (**C**) Western blot analysis of HIF-1α and phosphorylated p65 NF-κB levels in WT and ANT2-MKO BMDMs treated with or without H_2_O_2_ for 30 minutes. (**D**) mRNA expression of inflammatory genes in WT and ANT2-MKO BMDMs treated with or without H_2_O_2_ for 6 hours (*n* = 4 wells/group). (**E**) mRNA expression of inflammatory genes in WT and ANT2-MKO BMDMs transfected with mock or CA-HIF-1α–expressing plasmid vector. Forty-eight hours after transfection, cells were treated with or without PA or LPS for 8 hours (*n* = 2 wells/group). (**F**) Western blot analysis of mitophagic proteins in mitochondrial extracts purified from WT and ANT2-MKO BMDMs treated with or without PA or LPS in the presence or absence of MitoTEMPO for 4 hours. In all panels, all values are mean ± SEM. *P < 0.05; **P < 0.01; ***P < 0.001; ****P < 0.0001. NS, not significant. Statistical analysis was performed by 2-way ANOVA with Tukey’s multiple comparison test.

To assess whether the increase in mtROS is essential for ANT2-dependent proinflammatory macrophage activation, we first measured inflammatory signaling pathways in the presence or absence of a mitochondria-specific ROS scavenger, MitoTEMPO, in WT and ANT2-MKO BMDMs. As seen in Figure 8A and Supplemental Figure 8, A and B, MitoTEMPO treatment suppressed the enhanced PA- or LPS-induced HIF-1α expression and phosphorylated/activated p65 NF-κB levels in WT BMDMs, similar to the levels observed in ANT2-MKO BMDMs. Moreover, PA- or LPS-stimulated expression of proinflammatory genes, including *Tnf*, *Il6*, *Ccl2*, and *Ccl5*, was substantially attenuated by MitoTEMPO treatment, as they were in ANT2-MKO BMDMs (Figure 8B). MitoTEMPO treatment did not show additional effects in the ANT2-MKO BMDMs. On the other hand, induction of oxidative stress by hydrogen peroxide (H_2_O_2_) treatment induced HIF-1α and phosphorylated p65 NF-κB to comparable levels in WT and ANT2-MKO BMDMs (Figure 8C and Supplemental Figure 8, C and D). Moreover, H_2_O_2_ treatment induced mRNA expression of proinflammatory genes such as *Tnf*, *Il6*, *Hif1a*, and *Ccl5* to the same degree in WT and ANT2-MKO BMDMs (Figure 8D), suggesting that the increase in mtROS levels is necessary for ANT2-dependent proinflammatory macrophage activation. Next, we questioned whether the induction of HIF-1α is essential for ANT2-dependent proinflammatory gene expression. To address this question, we introduced a plasmid vector expressing a constitutively active form of HIF-1α (CA-HIF-1α) in WT and ANT2-MKO BMDMs, and measured proinflammatory gene expression. As seen in Figure 8E, overexpression of CA-HIF-1α partially restored PA- or LPS-induced expression of proinflammatory genes such as *Tnf*, *Il6*, *Ccl2*, and *Ccl5*. Lastly, we questioned whether the FFA- or LPS-stimulated ANT2-dependent mitophagy was due to increased mtROS levels. As seen in Figure 8, A and F, and Supplemental Figure 8, A, B, and E, PA or LPS induced a gradual increase in mitophagic protein expression, such as p62, LC3-II, Pink1, and Parkin, after 0.5–6 hours, and MitoTEMPO treatment inhibited this to levels comparable to those seen in ANT2-MKO BMDMs without MitoTEMPO treatment. These results suggest that ANT2-dependent increased mtROS production and mitochondrial damage stimulates mitophagy during proinflammatory macrophage activation.

## Discussion

Here, we demonstrate that myeloid lineage–specific ANT2 depletion improved insulin sensitivity and glucose tolerance in obesity. These beneficial effects were independent of changes in body weight, myeloid development, or macrophage growth and differentiation. Interestingly, the effects were associated with specific inhibition of obesity-induced monocyte recruitment into adipose tissue and proinflammatory ATM activation, blocking the development of metaflammation. At the molecular level, we found that the effect of ANT2 on macrophage biology can be traced to mitochondrial dysfunction. Specifically, we used a variety of inhibitors of specific mitochondrial functions to show that ANT2 MKO mimics the effect of these different inhibitors on mitochondrial performance. As an example of this, ANT2 MKO produced comparable effects to mtROS production and proinflammatory macrophage activation, as did mtPTP inhibitors, such as CsA and TRO19622. In addition, ANT2 MKO blocked both PA- and LPS-induced opening of the mtPTP, mtROS generation, and mitochondrial damage. ANT2 MKO also inhibited the effects PA and LPS to stimulate HIF-1α and proinflammatory gene expression. Taken together, these results indicate that ANT2-dependent opening of the mtPTP with subsequent increased mtROS levels are key triggering mechanisms underlying proinflammatory macrophage polarization and activation.

Increased mtROS production and mitochondrial damage play key roles in proinflammatory macrophage activation (75–77). Mechanistically, increased mtROS production increases cytosolic oxidized mtDNA fragments, which serve as ligands to activate the Nod-like receptor family pyrin domain–containing 3 (NLRP3) inflammasome (78, 79). Moreover, increased levels of mtROS stabilize HIF-1α, leading to greater HIF-1α protein expression, which drives M1-like macrophage polarization (37, 38). The mechanism for how TLR activation triggers mtROS generation and mitochondrial damage has not been understood. Here, we show that the TLR4 activators, PA and LPS, work through ANT2 to cause transient opening of the mtPTP, which induces mtROS generation, leading to mitochondrial dysfunction. Thus, our results provide a previously unknown mechanism for how mtROS generation and mitochondrial damage are triggered during proinflammatory macrophage activation.

We found that the expression of TLR2, TLR4, and TLR signaling genes was not changed by ANT2 depletion. On the other hand, LPS-indcued TRAF6 expression and mitochondrial translocation was diminished in ANT2-MKO BMDMs compared with WT BMDMs, along with decreases in LPS-induced JNK and NF-κB activation. Previously, it was shown that ROS increases TRAF6 (which is an E3 ubiquitin ligase) expression and autoubiquitination, facilitating increased TRAF6-TAK1 interaction, TAK1 ubiquitination, and TAK1 and JNK activation (80). Moreover, TRAF6 stimulates mtROS production by interacting with a mitochondrial protein, ECSIT, and facilitating its ubiquitination and accumulation in the mitochondrial periphery (81). Therefore, it is possible that ANT2-dependent mitochondrial ROS production and ROS-induced TRAF6 activation form a feed-forward activation loop, along with HIF-1α stabilization, to potentiate proinflammatory macrophage activation.

Although prolonged opening of the mtPTP typically leads to necrotic cell death (82, 83), transient opening occurs even in quiescent cells and serves as a signaling mechanism (84–86). For example, during early embryonic development in mice, opening of the mtPTP occurs in myocytes for days and limits mitochondrial maturation and myocyte differentiation until sufficient oxygen supply becomes available (87–89). Moreover, in adult mice, transient opening of the mtPTP and increased mtROS levels in cardiac myocytes are essential for the protective effects of ischemic preconditioning (90). ANTs are components of the mtPTP and exert modulatory roles in mtPTP opening (48, 49). For example, combinatorial deletion of ANT isoforms abolishes ANT ligand–induced mtPTP opening, and also increases the Ca^2+^ concentration threshold required to open the mtPTP. We find that *Ant2* is the predominant isoform expressed in macrophages, and obesity increases *Ant2* expression in ATMs. Importantly, depletion of ANT2 in macrophages substantially attenuated LPS- or PA-induced opening of the mtPTP. These results indicate a function of ANT2 to mediate FFA-induced opening of the mtPTP with subsequent mtROS generation, triggering proinflammatory macrophage activation.

Independent of mtPTP, ANT2 can directly regulate mitophagy (46), which also directly impacts proinflammatory macrophage activation. Previously, it was shown that mitophagy increases during proinflammatory macrophage activation. Moreover, obesity induces autophagy in ATMs through unknown mechanisms (91). We found that ANT2 depletion decreased PA- or LPS-induced mitophagy. Interestingly, inhibition of mtROS by MitoTEMPO treatment attenuated PA- or LPS-induced mitophagic protein expression in WT macrophages, comparable to the effect of ANT2 MKO in macrophages not treated with MitoTEMPO. Therefore, it is likely that the increase in mitophagy during proinflammatory macrophage activation is secondary to the ANT2-dependent increase in mtROS production, which causes mitochondrial damage. Thus, ANT2 can further relay the proinflammatory macrophage activation signals by directly mediating mitophagy.

In addition to this, it seems likely that ANT2-dependent increases in uncoupled respiration contribute to obesity-induced mitochondrial stress, enhancing proinflammatory ATM activation. Notably, the decrease in mitochondrial capacity during M1-like macrophage polarization was quite robust, resulting in an increase in the basal mitochondrial operation rate from approximately 45% to approximately 90%. This enhances electron flux along the mitochondrial electron transport chain, which should result in a further increase in premature electron escape to molecular oxygen (i.e., mtROS generation). Therefore, we suggest that FFA-stimulated ANT2-dependent increases in uncoupled macrophage mitochondrial respiration enhance mitochondrial stress. This effect of ANT2 appears to be unique, since overexpression of UCP2 blocks mtROS generation and *Ucp2*-KO macrophages generate more ROS (92). Therefore, the combinatorial effects of ANT2 on mtPTP opening and increased uncoupled mitochondrial respiration promote mitochondrial dysfunction, leading to proinflammatory ATM activation in obesity.

Recently, it was suggested that in addition to surface TLR activation by extracellular FFAs, intracellular FFAs can also play a role in obesity-induced proinflammatory ATM activation (25, 28–32). Several lines of evidence indicate that ANT2 could serve as an intracellular mitochondrial FFA sensor mediating FFA-induced uncoupled mitochondrial respiration and mtPTP opening (47, 50). In this study, we observed that ANT2 is necessary for PA-induced mtROS production and uncoupled mitochondrial respiration in macrophages.

Previously, we reported that early in the onset of obesity, increased FFAs stimulate adipocyte ANT2 and this triggers adipocyte chemokine production, with increased immune cell infiltration (51). In the current study, we demonstrate that increased FFAs can also promote propagation of adipose tissue inflammation by stimulating ANT2-dependent proinflammatory ATM activation. Adipocytes have relatively abundant mitochondria and ANT1 expression, and the effect of ANT2 was mainly mediated by increased FFA-induced uncoupled mitochondrial respiration. However, in macrophages, which have more limited mitochondrial capacity and ANT1 expression, we find that the effects of ANT2 were mainly mediated by opening of the mtPTP, causing increased mtROS production and mitochondrial damage.

In summary, we report a mechanism for how obesity induces proinflammatory macrophage activation through FFA-stimulated ANT2 effects. *Ant2* expression was increased in ATMs of obese mice and the depletion of myeloid ANT2 blocked obesity-induced proinflammatory ATM activation, without affecting body weight or myeloid development or growth. In vitro studies revealed that ANT2 mediates FFA-induced transient opening of the mtPTP, causing increased mtROS production and proinflammatory macrophage activation. Depletion of myeloid ANT2 was sufficient to improve adipose tissue inflammation, insulin resistance, and glucose intolerance in obesity. The current studies identify a component of the mechanism underlying the propagation of metaflammation, which could lead to new opportunities for therapeutic intervention.

## Methods

### Generation of myeloid lineage–specific ANT2-KO mice.

To generate myeloid lineage–specific ANT2-KO mice (*LysM-Cre*^+/–^ *Slc25a5*/*Ant2^fl/Y^*), mice with loxP recombinase recognition site–flanked *Slc25a5*/*Ant2* (*Ant2^fl/Y^* hemizygous male and *Ant2^fl/fl^* homozygous female) (53) were bred with mice expressing Cre recombinase driven by the LysM promoter. Mice were housed in colony cages under 12-hour light/12-hour dark cycles. For HFD studies, mice were subjected to 60% HFD for 18 weeks or for the indicated time periods.

### Tolerance tests.

For oral glucose tolerance tests (OGTTs), the mice were fasted for 6 hours and basal blood samples were taken, followed by oral glucose gavage (2 g/kg). Blood samples were drawn at 10, 20, 30, 45, 60, 90, and 120 minutes after gavage. For insulin tolerance tests (ITTs), mice were fasted for 6 hours, and basal blood samples were taken, followed by intraperitoneal injection of insulin (0.35 and 0.6 U/kg for NCD- and HFD-fed mice, respectively). HOMA-IR was calculated as described previously (93, 94).

### ATM preparation and analysis.

ATM preparation and flow cytometry analysis/sorting were performed as described previously (51). Briefly, eWAT was minced and digested in buffered collagenase (1 mg/mL; Sigma-Aldrich) for 30 minutes at 37°C with shaking. The digests were filtered through 70 μm cell strainers and centrifuged at 500*g* for 5 minutes. Pellets were incubated in RBC lysis buffer (eBioscience; detailed information for all materials used in the current study can be found in Supplemental Table 2) for 5 minutes, washed twice with PBS, and resuspended into PBS supplemented with 1% endotoxin-low bovine serum albumin (BSA). For flow cytometry analysis or cell sorting, cells were incubated with anti-CD16/anti-CD32 blocking antibodies for 15 minutes, and then stained with the fixable Live/Dead Aqua staining dye and different combinations of specific antibodies conjugated with fluorescent dyes, as indicated in the figure legends. Cells were gently washed twice in ice-cold PBS and resuspended in PBS supplemented with 1% endotoxin-low BSA. For intracellular staining (e.g., Ki67 or active caspase-3), cells were fixed and permeabilized using the Foxp3 Staining Kit according to the manufacturer’s instructions. Stained cell samples were measured in the BD FACSCanto (Orange RUO) or sorted in a BD FACSAria II. The data were analyzed using FlowJo 10.6.1 software (Tree Star).

### Single-cell preparations from mouse mesenteric lymph nodes and spleen.

Spleens and mesenteric lymph nodes were collected from WT and ANT2-MKO mice, and minced through 70 μm mesh filters into conical tubes using a syringe plunger. The filtered cells were centrifuged at 500*g* for 5 minutes at 4°C, washed twice, and analyzed by flow cytometry.

### Insulin-stimulated AKT phosphorylation in mouse tissues.

In vivo tissue insulin action was evaluated by measuring insulin-stimulated Akt phosphorylation (Ser473) in liver, skeletal muscle (quadriceps), and eWAT. Briefly, after 6-hour fasting, mice were anesthetized and parts of these insulin target tissues were collected to measure basal levels of Akt phosphorylation. After a dose of insulin (0.6 U/kg body weight) was injected via the portal vein, parts of the liver, skeletal muscle, and eWAT were collected at 2, 5, and 10 minutes. Total and phosphorylated Akt levels were measured by Western blot analyses.

### BMDM preparation.

Bone marrow cells from were isolated from femurs of WT or ANT2-MKO mice. Cells were treated with RBC lysis buffer (eBioscience) to remove red blood cells for 5 minutes, centrifuged at 400*g* for 5 minutes, and resuspended in BMDM culture medium (RPMI-1640 supplemented with 10% heat-inactivated fetal bovine serum (FBS), 100 U/mL penicillin, and 100 mg/mL streptomycin. To induce macrophage differentiation, 20 ng/mL M-CSF) (Biolegend, catalog 576406) was added to the culture media and maintained at 37°C in a humidified 5% CO_2_ incubator for 7 days. The media were changed once on day 3 of differentiation. One day prior to cell harvesting, cells were replated in plates/dishes of the appropriate size for each experiment. To drive M1-like macrophage polarization, 7-day-differentiated BMDMs were incubated in the presence of 600 ng/mL LPS for 24 hours.

### HIF-1α overexpression.

For HIF-1α overexpression studies, differentiated BMDMs were transfected with control plasmid or CA-HIF-1α–expression plasmid vector using *Trans*IT-LT1 Transfection Reagent (Mirus Bio, catalog MIR2300). Six hours after transfection, the medium was replaced with fresh medium. Forty-eight to 60 hours after transfection, cells were stimulated with PA (250 μM) or LPS (600 ng/mL) for the indicated periods of time.

### Measurement of intracellular ROS and mtROS.

Cytosolic ROS and mtROS levels were measured using 5,6-chloromethyl-2′,7′-dichlorodihydro-fluorescein diacetate (CM-H2DCF-DA; Thermo Fisher Scientific, catalog C6827) and MitoSOX red (Thermo Fisher Scientific, catalog M36008) fluorescent dyes, respectively. Thirty minutes after dye loading, cells were treated with LPS or PA for the periods of time indicated in the figure legends.

### Western blot analysis.

Tissues or cell samples were lysed in ice-cold IP buffer (50 mM pH8.0 Tris, 150 mM NaCl, 1 mM EGTA, 1 mM EDTA, 1% Triton X-100, 10% glycerol) containing EDTA-free protease and phosphatase inhibitor mixture (Roche Diagnostics) and centrifuged at 15,000*g* and 4°C for 20 minutes. The supernatants were separated in SDS-PAGE gels (Bio-Rad) and electrotransferred to polyvinylidene difluoride membranes. The membranes were blocked for 1 hour in Tris-buffered saline supplemented with Tween 20 (TBS-T; 10 mM Tris-HCl pH 7.4, 150 mM NaCl, 0.1% Tween 20) and 5% BSA, and then incubated with specific antibodies indicated in the figures at 4°C overnight. After 3 washes with fresh TBS-T for 15 minutes each, the membranes were incubated with secondary anti-rabbit or -mouse IgG conjugated with horseradish peroxidase (Jackson ImmunoResearch Laboratories; 1:5,000 dilution) and visualized using the ECL system (Thermo Fisher Scientific or Millipore) followed by autoradiography or the Bio-Rad ChemiDoc XRS+ imaging system. Intensity of the bands in the autoradiograms was measured using ImageJ software (NIH).

### Quantitative real-time RT-PCR.

Total RNA was extracted with TRIzol reagent (Invitrogen) or RNeasy mini kit (Qiagen). Synthesis of cDNA was performed using the High-Capacity cDNA Reverse Transcription kit (Applied Biosystems). Quantitative real-time PCR was performed using Power SYBR Green PCR Master Mix (Applied Biosystems) and the primers listed in Supplemental Table 3.

### mtDNA content.

Total DNA was extracted from cells using a proteinase K DNA extraction method. The relative mtDNA copy number was determined by normalizing mtDNA copy number to nuclear DNA (*18S* rRNA) copy number. Primer sequences are shown in Supplemental Table 3.

### CS activity.

CS activity was measured using a commercial kit according to the manufacturer’s instructions (BioVision).

### In vivo monocyte tracking experiments.

In vivo monocyte tracking experiments were performed as described previously, with minor modifications (51). PBMCs were isolated using a density gradient centrifugation method with Histopaque-1077 (Sigma-Aldrich, catalog 10771), according to the manufacturer’s instructions. Isolated PBMCs were washed once in serum-free medium (RPMI-1640) and suspended in 2 mL diluent solution C (included in the PKH67 labeling kit). A total of 2 mL of 2 mM PKH67 (Sigma-Aldrich, catalog MINI67-1KT) in diluent C was added and mixed, and the cells were incubated for 10 minutes at room temperature in the dark. The staining reaction was halted by the addition of an equal volume (2 mL) of medium supplemented with 10% FBS. The mixture was centrifuged, and the cells were washed once and resuspended in serum-containing medium. The PKH67-labeled PBMCs were counted and approximately 2 × 10^6^ viable cells were suspended in 0.2 mL PBS and injected into the tail vein of each of the WT or ANT2-MKO mice. Three days after injection, SVCs were isolated from eWAT and analyzed by flow cytometry.

### Transmission electron microscopy.

Cells were fixed with freshly prepared fixative containing 2.5% glutaraldehyde in 0.15 M cacodylate buffer. Fixed cells were postfixed in 1% OsO_4_ in 0.1 M cacodylate buffer for 1 hour on ice. Cells were stained en bloc with 2%–3% uranyl acetate for 1 hour on ice as described previously (95). The cells were dehydrated in a graded series of ethanol (20%–100%) on ice followed by 1 wash with 100% ethanol and 2 washes with acetone (15 minutes each) and embedded with Durcupan. Ultrathin (50–60 nm) sections were cut on a Leica UCT ultramicrotome, and picked up on Formvar and carbon-coated copper grids. Sections were stained with 2% uranyl acetate for 5 minutes and Sato’s lead stain for 1 minute. Grids were viewed using a JEOL JEM1400-plus TEM and photographed using a Gatan OneView digital camera with 4k × 4k resolution. Morphometric measurements were made randomly on deidentified samples. The free-hand tool in ImageJ was used to determine mitochondrial area by manually tracing around the mitochondrial outer membrane, as described previously (96, 97). The area of each crista membrane was also calculated in the same manner. The sum of the areas of the total complement of cristae was then divided by the sum of the mitochondrial area to obtain the cristae density.

### Morphometric analysis of autophagy.

Autophagy was analyzed based on cell organelle structures and the quantity of autophagic vacuoles. Briefly, autophagic vacuoles were counted as autophagosomes when they met 2 or more of the following criteria: double membranes (complete or at least partially visible), absence of ribosomes attached to the cytosolic side of the membrane, luminal density similar to the cytosol, and identifiable organelles or regions of organelles in their lumens. Vesicles of similar size but with a single membrane (or less than 40% of the membrane visible as double), luminal density lower than the surrounding cytosol, and multiple single-membrane-limited vesicles containing light or dense amorphous material were classified as autophagosomes. For morphometric purposes, both types of autophagic vesicles were pooled together in this study. To determine autophagy density per cell, the sum of autophagosome areas was divided by the cell area and multiplied by 100.

### mKeima-Red mitophagy analysis.

To determine quantitative changes in mitophagy, BMDMs were transfected with mKeima-Red-Mito-7 (Addgene, plasmid 56018). The excitation spectrum of mKeima-Red shifts from 440 to 620 nm when mitochondria are delivered from the cytosol to acidic lysosomes, which occurs during mitophagy (98, 99). Thirty-six hours after transfection, cell were treated with 100 μM PA or 600 ng/mL LPS for 18 hours and cell images were acquired using the JuLI Stage (NanoEnTek) imaging system with 458 nm (green, mitochondria at neutral pH) and 561 nm (red, mitochondria under acidic pH) lasers. Images were analyzed using ImageJ software.

### Opening of the mtPTP.

Opening of the mtPTP was measured using a Mitoprobe Transition Pore Assay Kit (Thermo Fisher Scientific). Briefly, cells were loaded with a colorless dye, calcein AM, which is cleaved by intracellular esterases to produce the polar fluorescent dye calcein. By adding CoCl_2_, cytosolic calcein was quenched while mitochondrial calcein remain fluorescent. Cells were then treated with or without PA (100 μM) or LPS (600 ng/mL). Five minutes later, leakage of calcein trapped in mitochondria into the cytosol by the opening of the mtPTP was measured as a function of decreased calcein signal in the cells.

### Efferocytosis assay.

In vitro efferocytosis assays were performed by coculturing BMDMs with apoptotic hepatocytes labeled with carboxyfluorescein succinimidyl ester (CFSE) at a ratio of 3 apoptotic hepatocytes per macrophage for 24 hours. Hepatocytes that had not been phagocytosed were removed by washing the wells with PBS 3 times. Efferocytosis by adherent macrophages was assessed by imaging with JuLi Stage and the results are expressed as mean florescence intensity of CFSE in the seeded macrophage areas. To induce apoptosis, hepatocytes were incubated with 1 μM staurosporine for 1 hour at 37°C with 5% CO_2_ after staining with CFSE.

### RNA-seq data analysis.

Quality control of the raw fastq files was performed using the software tool FastQC v0.11.8 (http://www.bioinformatics.babraham.ac.uk/projects/fastqc). Sequencing reads were trimmed with Trimmomatic v0.38 (100) and aligned to the mouse genome (GRCm38_p6) using the STAR aligner v2.5.1a (101). Read quantification was performed with RSEM v1.3.0 (101) and Ensembl release 98 (102). The R BioConductor packages edgeR (103) and limma (104) were used to implement the limma-voom (105) method for differential expression analysis. In brief, genes with low expression — those not having counts per million (cpm) ≥ 1 in at least 2 of the samples — were filtered out and then trimmed mean of M values (TMM) (106) normalization was applied. The experimental design was modeled upon condition (~0+ condition). The voom method was employed to model the mean-variance relationship in the log(cpm) values, after which lmFit was used to fit per-gene linear models and empirical Bayes moderation was applied with the eBayes function. Significance was defined by using an adjusted *P*-value cutoff of 0.05 after multiple testing correction (107) using a moderated *t* statistic in limma. Functional enrichment of the differentially expressed genes was performed using GSEA (108).

### Quantification and statistics.

All statistical parameters, including the number of replicates (*n*), can be found in the figure legends. Statistical analyses were performed using Graph Pad Prism 8 software or Microsoft Excel. Data are presented as the mean ± SEM. Individual pairwise comparisons were performed using 2-tailed Student’s *t* test. For experiments involving 2 or more factors, data were analyzed by 1-way repeated measures ANOVA followed by Dunnett post hoc multiple comparison tests. Data were assumed to follow a normal distribution (no tests were performed). A *P* value of less than 0.05 was considered significant: *P < 0.05; **P < 0.01; ***P < 0.001; ****P < 0.0001.

### Study approval.

All animal procedures were approved by the Institutional Animal Care and Use Committee of University of California, San Diego.

## Author contributions

JSM performed the majority of experiments and wrote the manuscript. FFDC, JYH, and JL supported flow cytometry analysis and/or BMDM experiments. FCGR supported in vivo experiments. AYA performed OCR and mitochondrial measurements. SKM performed electron microscopy analysis. CAN performed RNA-seq data analysis. YSL conceived, designed, and supervised the project, acquired funding, and wrote the manuscript. All authors discussed the results and commented on the manuscript.

## Supplementary Material

Supplemental data

## Version 1 10/22/2021

Electronic publication
