# Supplementary material for: ANT2 drives proinflammatory macrophage activation in obesity
Source: JCI Insight. 2021 Oct 22;6(20):e147033. doi: 10.1172/jci.insight.147033 (PMC8564915; doi:10.1172/jci.insight.147033)

Supplementary Figure Legends

Supplementary Figure 1. Generation of myeloid-specific ANT2 KO mice. (**A**) mRNA expression of *Ant1* and *Ant2* in bone marrow-derived monocytes of WT and ANT2 MKO mice (n=4 mice/group). (**B**) Ant2 mRNA expression in bone marrow-derived monocytes isolated from WT and ANT2 MKO mice during M-CSF-induced macrophage differentiation (n=3 mice/group). (**C**) mRNA expression of *Ant1* and *Ant2* in BMDMs of WT and ANT2 MKO mice (n=4 wells/group). (**D**) *Ant2* mRNA expression in eWAT (left; n=6 mice/group) and primary adipocytes (middle; n=6 mice/group) and SVCs (right; n=3 mice/group) isolated from eWAT of WT and ANT2 MKO mice. (**E**) mRNA expression of *Ant1* and *Ant2* in ATMs isolated from eWAT of WT and ANT2 MKO mice (n=4 mice/group). (**F**) mRNA expression of *Ant1* and *Ant2* in whole brain of WT and ANT2 MKO mice (n=6 mice/group). (**G**) Semi-quantitative PCR analysis of *Ant2* exon2 and 3 deletion in genomic DNAs purified from whole brain of WT and ANT2 MKO mice. Schematic diagram of the genomic DNA PCR strategy is illustrated on top. (**H**) Body weight of NCD WT and ANT2 MKO mice (n=5 WT and 5 KO mice). (**I**) eWAT Mass of NCD WT and ANT2 MKO mice (n=5 WT and 5 KO mice). (**J**) Average adipocyte size in eWAT of WT and ANT2 MKO mice (n=5 WT and 5 KO mice). (**K**) Liver Mass of NCD WT and ANT2 MKO mice (n=5 WT and 5 KO mice). (**L**) H & E staining of eWAT sections of NCD WT and ANT2 MKO mice. eWAT samples harvested from 5 WT and 5 KO individual mice were analyzed and representative pictures are shown. (**M**) H & E staining of liver sections of NCD WT and ANT2 MKO mice. Representative pictures are shown. eWAT samples harvested from 5 WT and 5 KO individual mice were analyzed and representative pictures are shown. (**N**) OGTT in NCD WT and ANT2 MKO mice (n=6 WT and 8 KO mice). (**O**) Fasting (6h) plasma insulin levels in NCD WT and ANT2 MKO mice (n=6 WT and 8 KO mice). (**P**) Body weight changes after HFD in WT and ANT2

MKO NCD WT and ANT2 MKO mice (n=6 WT and 8 KO mice). *P<0.05; **P<0.01; ***P<0.001; all error bars represent SEM.

Supplementary Figure 2. Insulin-stimulated Akt phosphorylation in liver, skeletal muscle, and eWAT of HFD WT and ANT2 MKO mice. (**A**) Western blot analysis of total and phosphorylated (Ser473) Akt was performed in total lysates of liver, skeletal muscle (quadriceps), and eWAT of HFD WT and ANT2 MKO mice. (**B**) Relative band intensity in panel A was measured, plotted, and presented.

Supplementary Figure 3. ANT2 depletion does not affect myeloid development or macrophage differentiation, and obesity induced increased *Ant2* expression in ATMs. (**A-B**) Flow cytometry analysis of the time course changes in macrophage differentiation of hematopoietic stem cells isolated from WT and ANT2 KO mice, stimulated by M-CSF *in vitro* (A). The proportion of $CD11b^+$ $F4/80^+$ macrophages during the course of BMDM differentiation is plotted in panel B (n=3 wells/time point). (**C-E**) Flow cytometry analysis of myeloid cells in spleen (C-left and E) and mesenteric lymph node (C-right and D) of WT and ANT2 MKO mice. The proportion of each of myeloid subsets is plotted in panel C (n= 5 mice/group). (**F**) Gating strategy for the flow cytometry analysis in Figure 2C. (**G-H**) Flow cytometry analysis of different $CD4^+$ T cell subsets in HFD WT and ANT2 MKO mice (n= 7 mice per group). (G) The proportion of different $CD4^+$ T cell subsets among total SVCs are plotted and presented. (H) Gating strategy for different $CD4^+$ T cell subsets. (**I-N**) Single cell RNA-seq analysis of *Ant2* expression in different subsets of adipose tissue macrophages and monocytes. UMAP projections of identified graph-based adipose tissue macrophage (I) and monocyte (J) clusters from scRNA-seq data derived from *CD45*$^+$ cells sorted from the stromal vascular fraction of eWATs of NCD and HFD WT mice are shown in

panels of I and J. k-nearest neighbor graph algorithm was applied to identify 6 clusters of adipose tissue macrophages and monocytes as illustrated and color-coded in panel I. Distribution and expression levels of *Ant2* in each of the adipose tissue macrophages (L) and monocytes (N) are presented as heat maps in panels L and N. Expression levels of *Ant2* in different ATM subsets expressing *Itgax*$^+$ (encoding CD11c) and *Cd9*$^+$ are plotted in a bar graph (M). n= 4 NCD and 4 HFD (18 weeks) mice. (**O**) *Ant1* and *Ant2* mRNA expression in WT BMDMs treated with or without LPS or IL-4/IL-13 for 24h (n=4 wells/group). (**P**) mRNA expression of *Ant1* and *Ant1* in ATMs isolated from NCD and HFD WT mice (n=4 mice/group). *P < 0.05, **P < 0.01; all error bars represent SEM.

Supplementary Figure 4. Analysis of WT and ANT2 KO BMDMs. (**A**) Gating strategy for the flow cytometry analysis of BMDMs treated with or without LPS. (**B**) Quantitative changes in TLR2, TLR4, and TRAF6 expression relative to actin expression in Figure 4F Western blot images were calculated and plotted. (**C**) mRNA expression of TLRs and downstream signaling molecules in WT and ANT2 KO BMDMs (n=4 wells/group). (**D**) Western blot analysis of TRAF6 expression in the cytoplasmic and mitochondrial fraction of WT and ANT2 KO BMDMs treated with or without for 6 h. (**E**) Quantitative changes in the protein expression relative to GAPDH expression in panel D images were calculated and plotted.

Supplementary Figure 5. Gating strategies for flow cytometry analysis of ATMs. (**A**) Gating strategy for flow cytometry analysis of ATMs in Figure 5B. (**B**) mRNA expression of chemokine receptor genes in WT and ANT2 KO monocytes. *P < 0.05, **P < 0.01; all error bars represent SEM. (**C**) Gating strategy for flow cytometry analysis of ATMs in Figures 5C, 5D, and 5E.

Supplementary Figure 6. OCR, PGC1α expression, and mitophagy in WT and ANT2 KO BMDMs treated with or without LPS or PA. (**A**) Basal OCR was measured in WT and ANT2 KO BMDMs treated with or without LPS for 24h, in the presence or absence of acute PA treatment (n= 5 wells/group). (**B**) Oligomycin-insensitive (uncoupled) OCR in WT and ANT2 KO BMDMs treated with or without LPS for 24h, in the presence or absence of acute PA treatment (n= 5 wells/group). (**C**) PGC-1α expression in WT and ANT2 KO BMDMs treated with PA or LPS for 24h. (**D**) Quantitative measurement of mitophagy using mtKeima-red in WT and ANT2 KO BMDMs treated with or without LPS for 24. Representative pictures are shown on the left. The ratio between red and green fluorescence was calaculated and ploted on the right. (**E**) Electron microscopy analysis of autophagosome formation in WT and ANT2 KO BMDMs treated with or without PA or LPS for 24h. Representative pictures are shown on the left. The number of autophagosome in a given cellular area is plotted on the right. *P < 0.05, **P < 0.01, ***P<0.001; all error bars represent SEM.

Supplementary Figure 7. Relative band intensity in the Western blot images in Figure 7D.

Supplementary Figure 8. Inflammatory and mitophagic protein expression in WT and ANT2 KO BMDMs. (**A**) Relative band intensity of the Western blot images in Figure 8A. (**B**) Western blot analysis of inflammatory and mitophagic protein expression in WT and ANT2 KO BMDMs treated with or without PA or LPS, in the presence or absence of MitoTEMPO for 30 min. (**C**) Relative band intensity of the Western blot images in Figure 8C. (**D**) Western

blot analysis of HIF-1α in WT and ANT2 KO BMDMs treated with or without H_2O_2 for 1 or 4h.

(**E**) Relative band intensity of the Western blot images in Figure 8F.

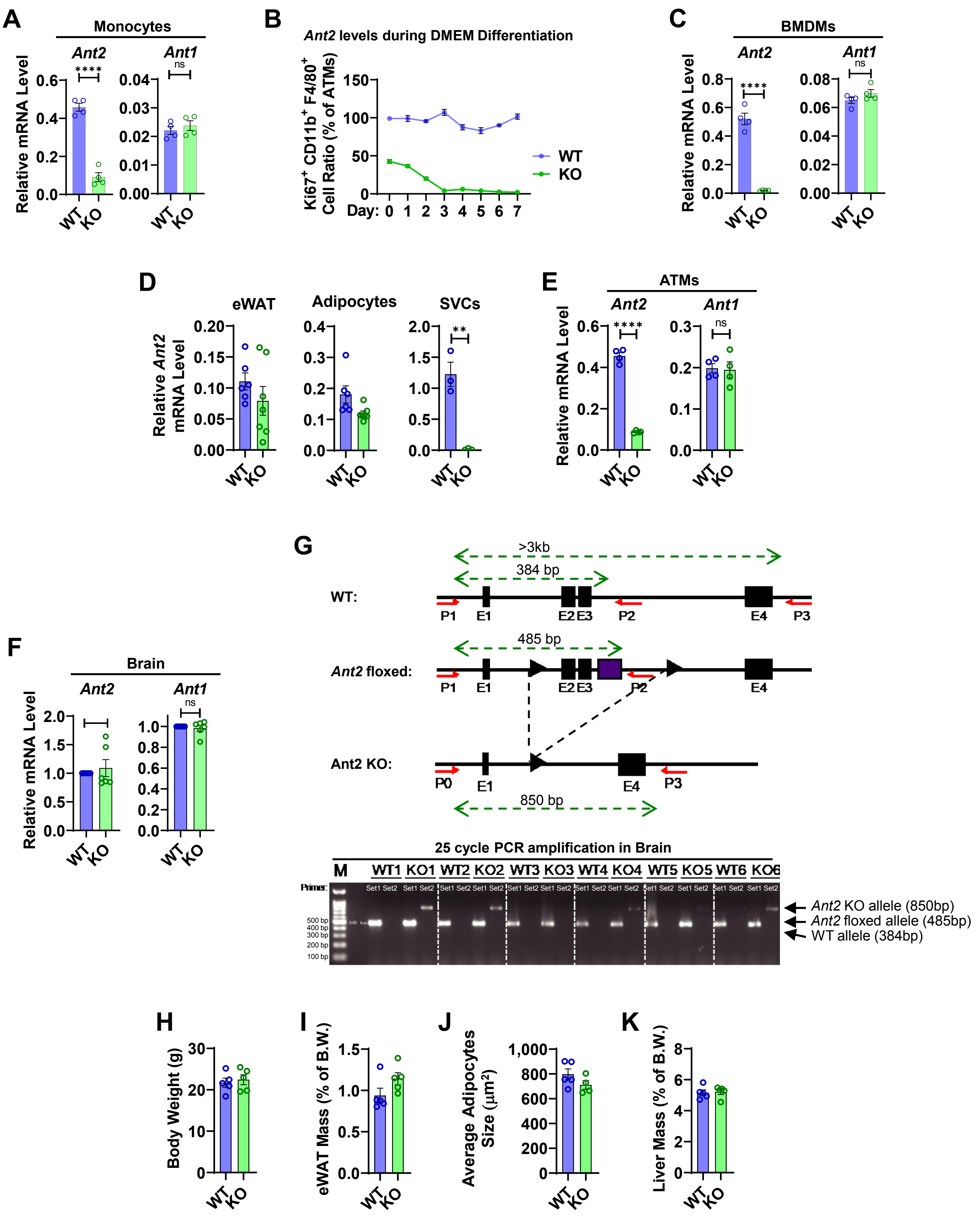

Supplementary Figure 1

L

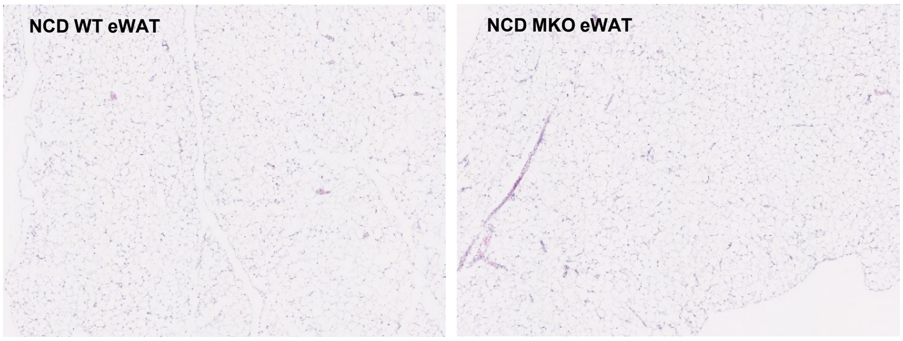

M

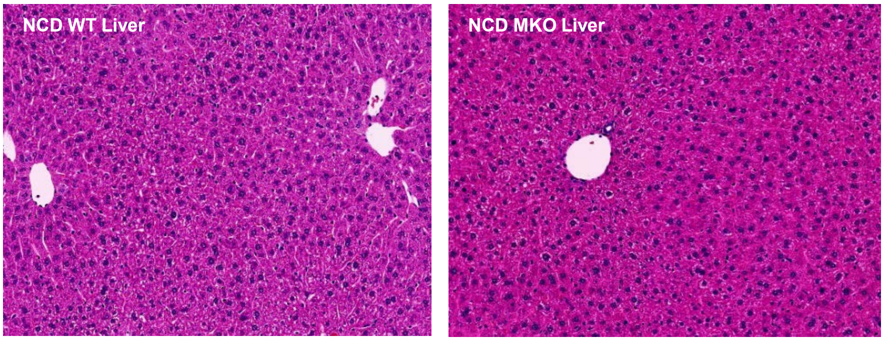

N

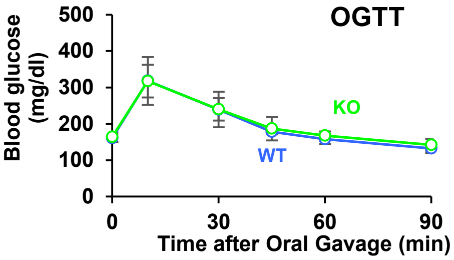

O

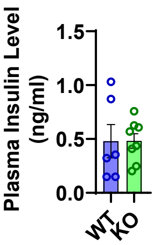

P

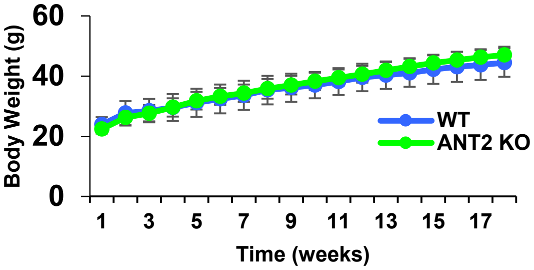

Supplementary Figure 1

A

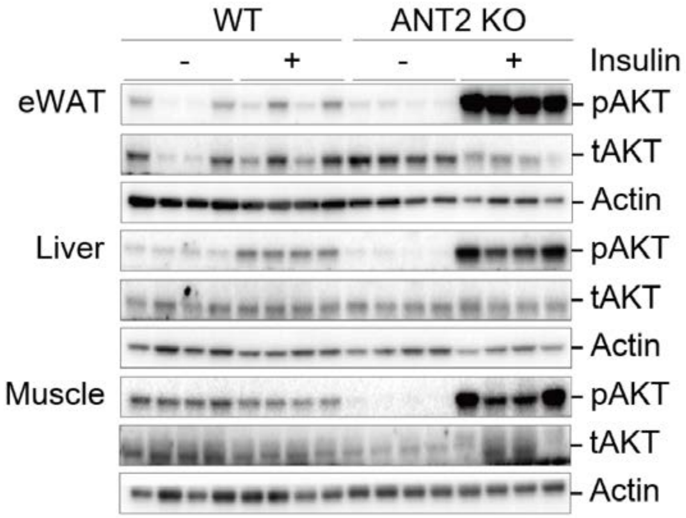

B

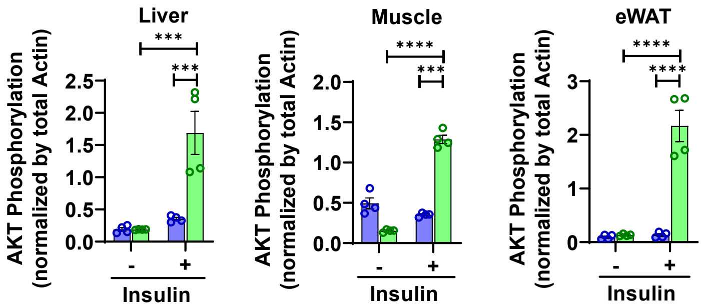

Supplementary Figure 2

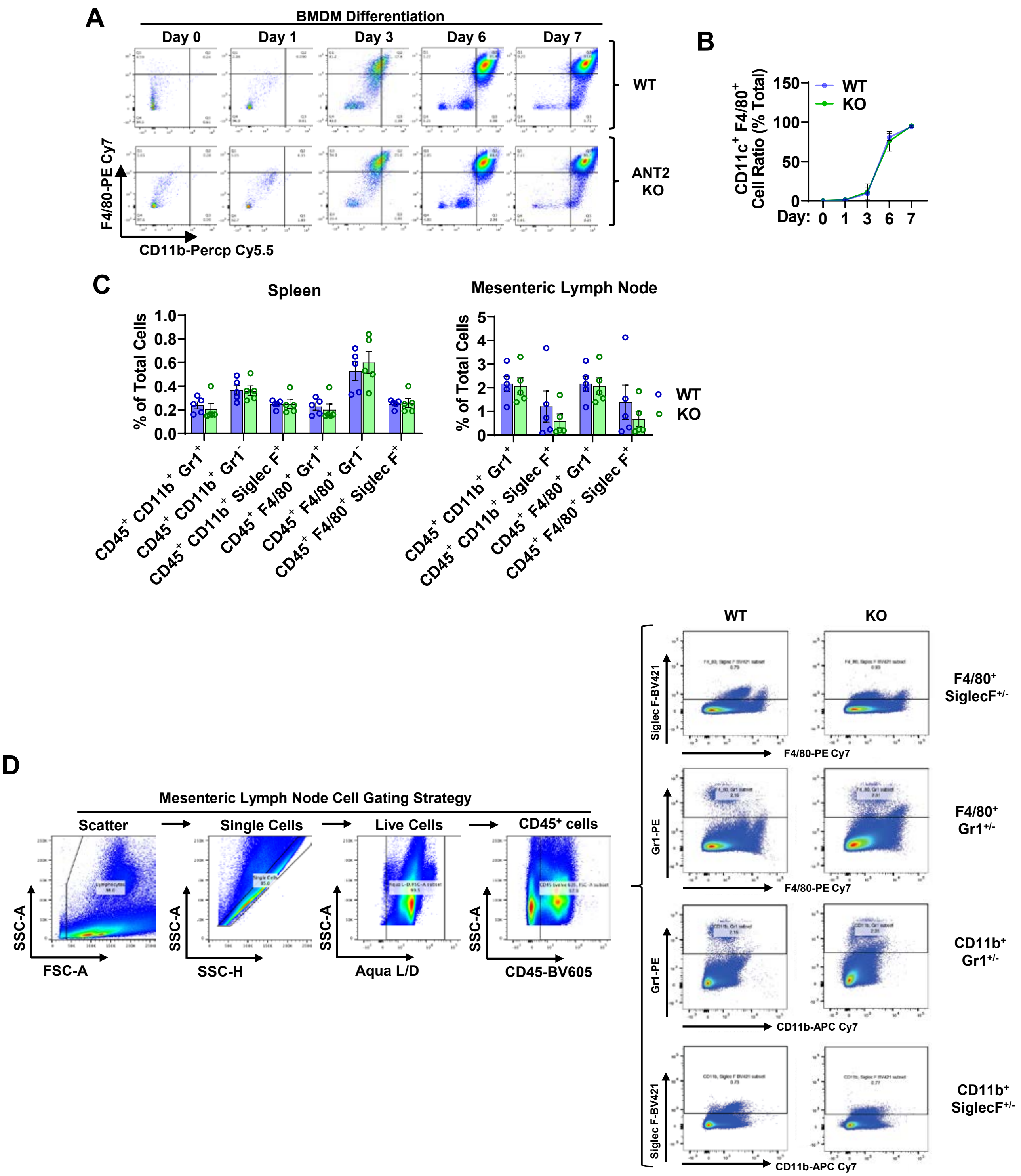

Supplementary Figure 3

E

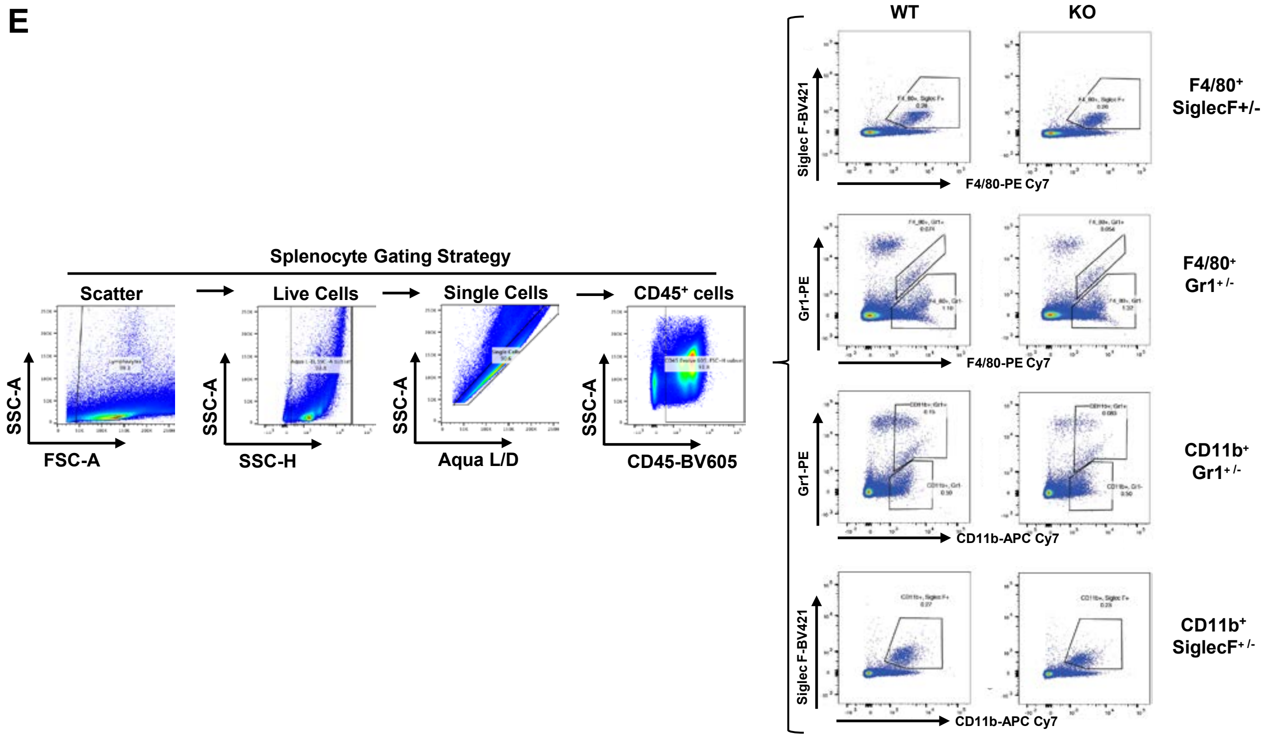

F

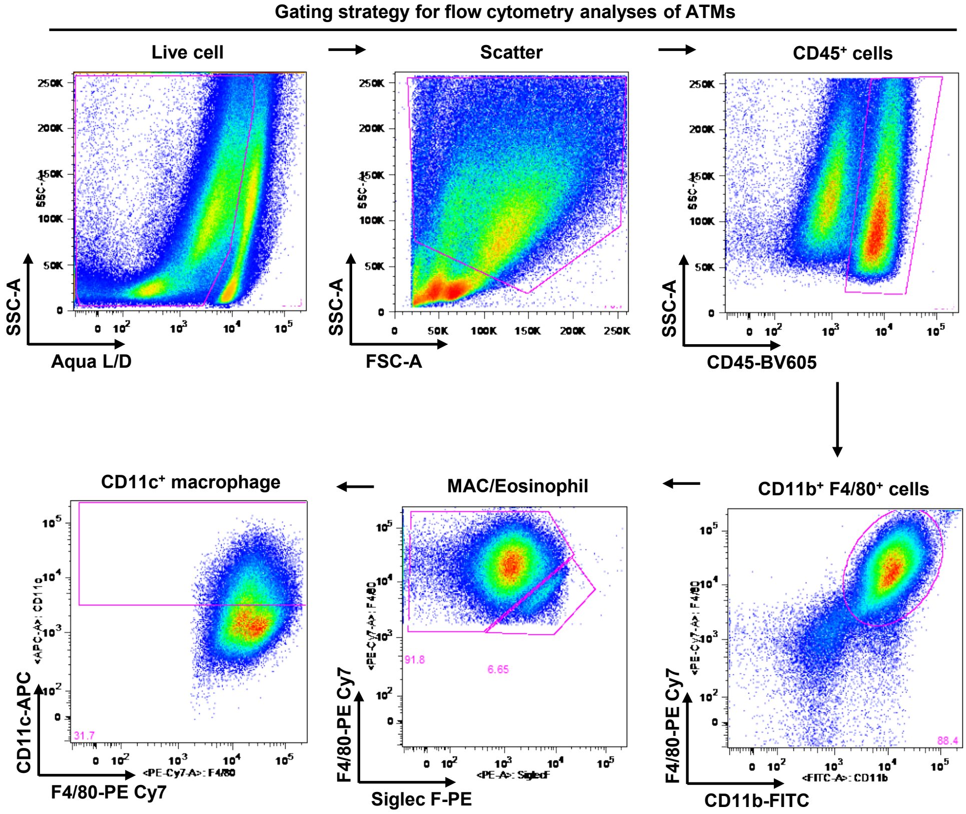

Supplementary Figure 3

G

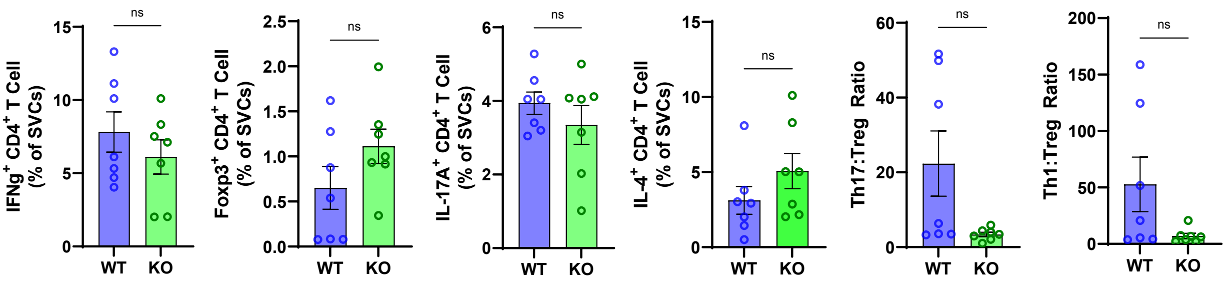

H

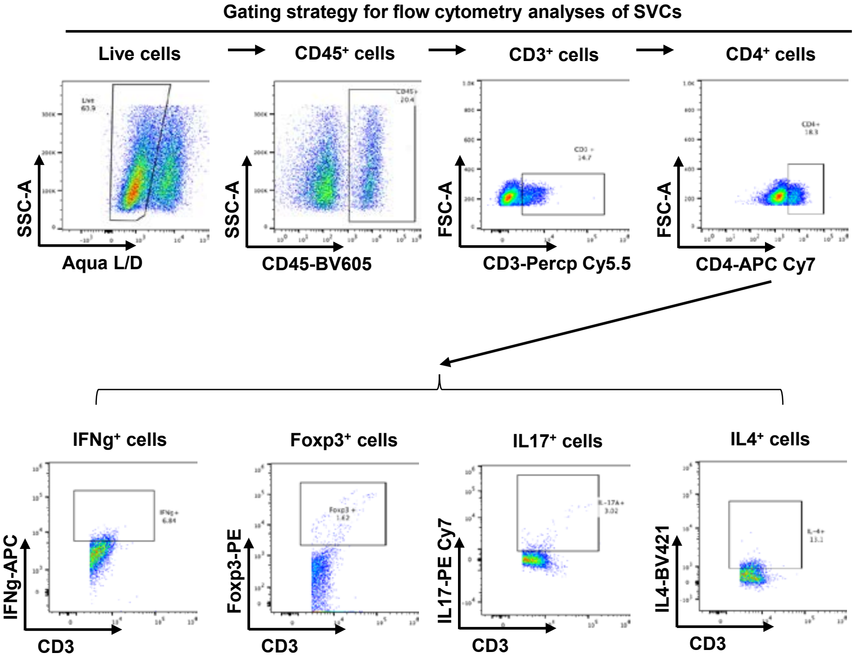

Supplementary Figure 3

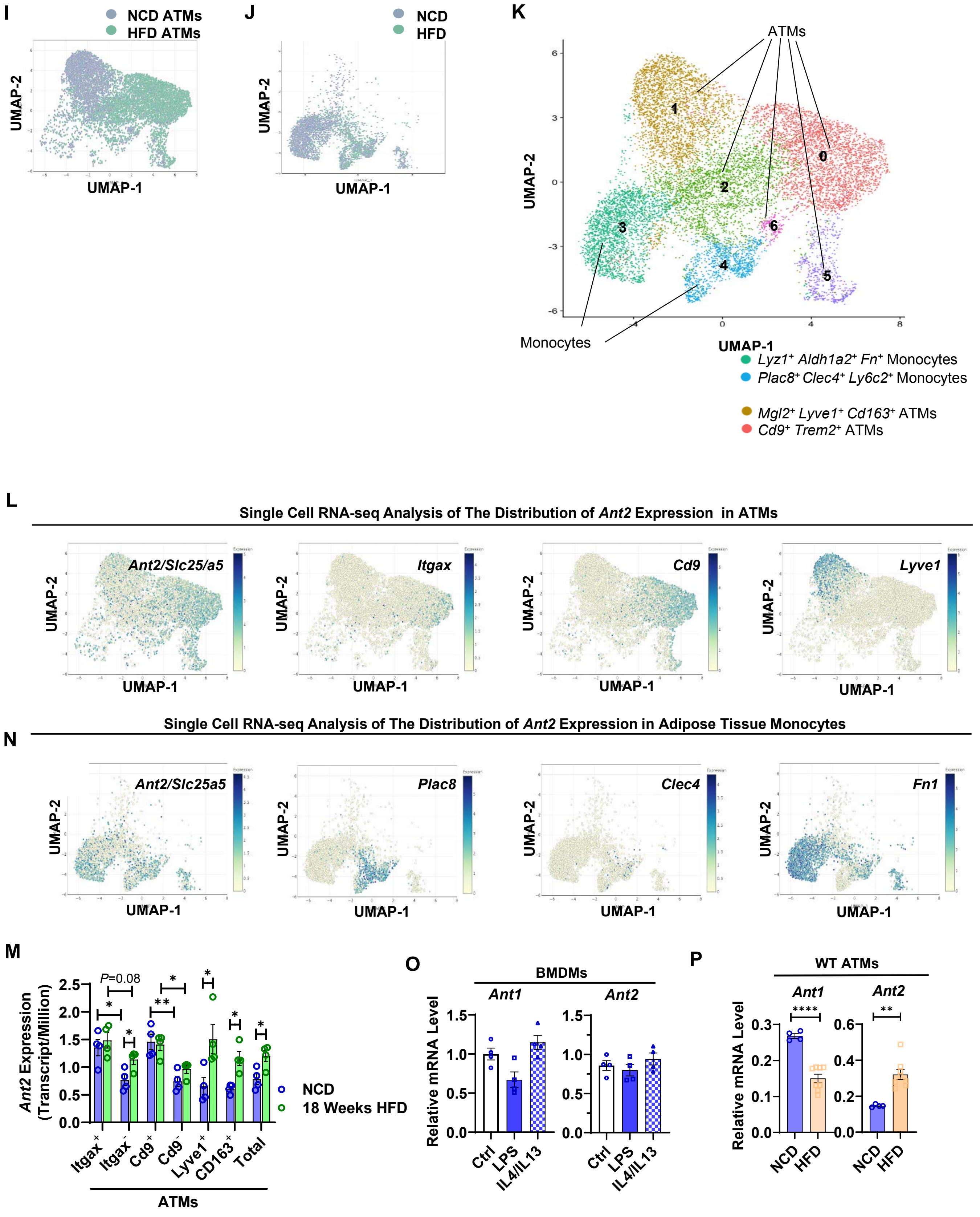

Supplementary Figure 3

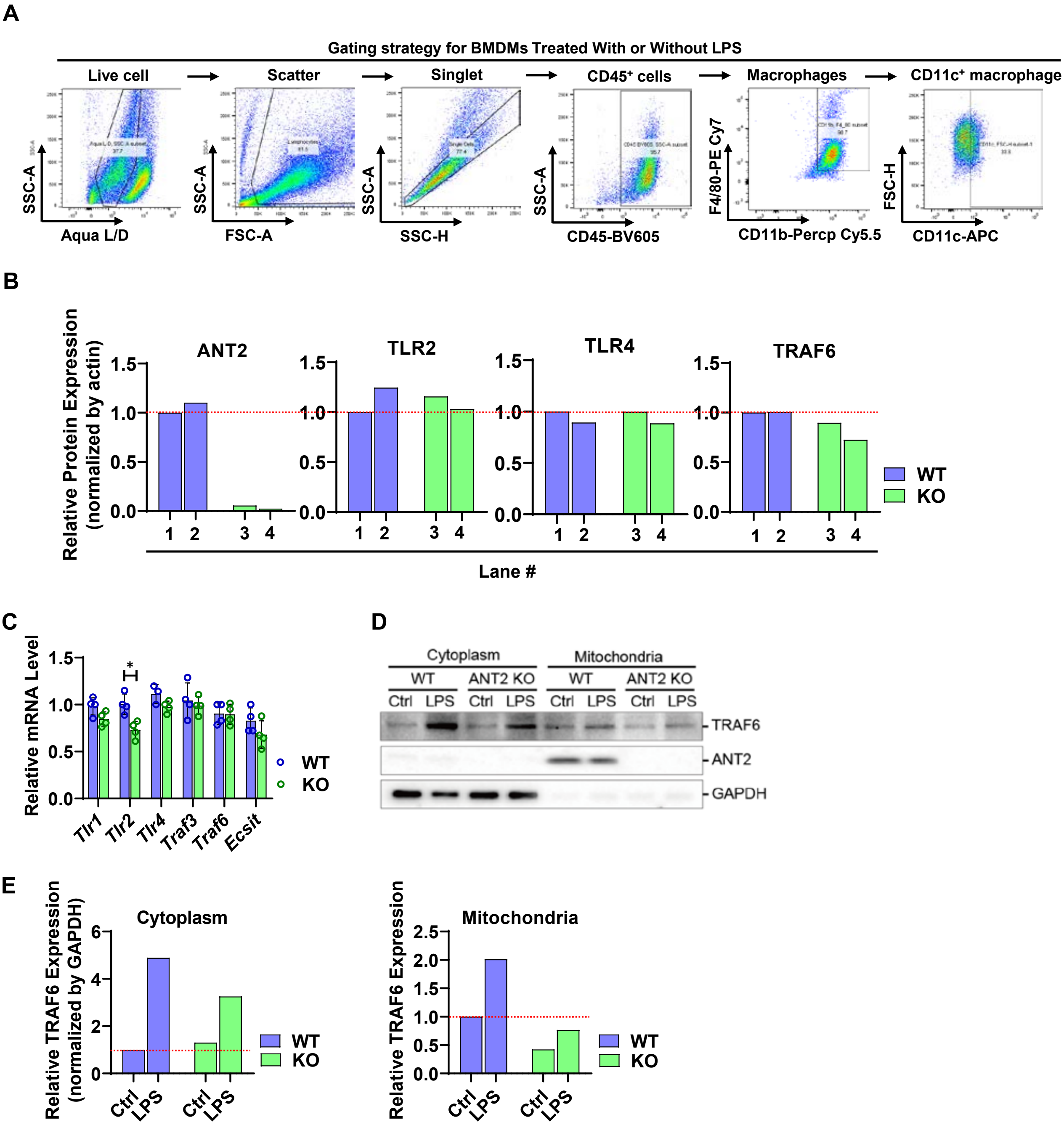

Supplementary Figure 4

A

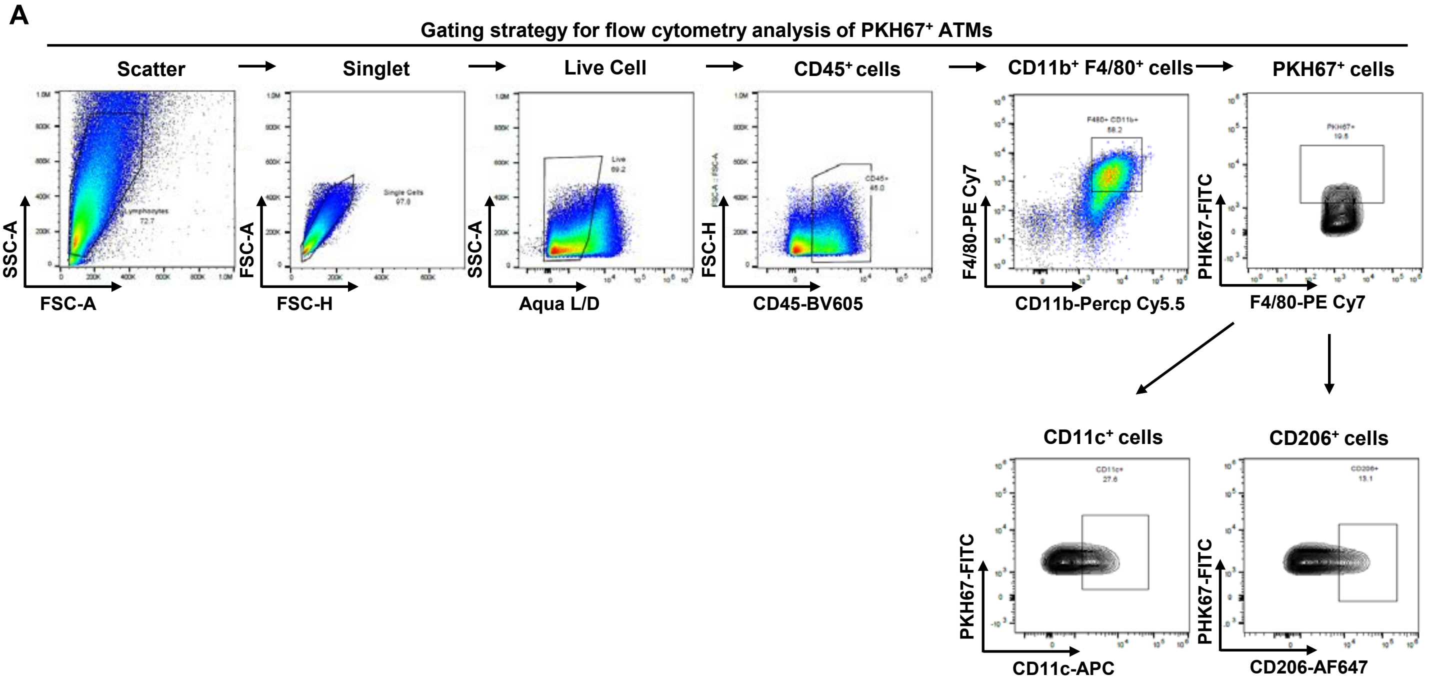

B

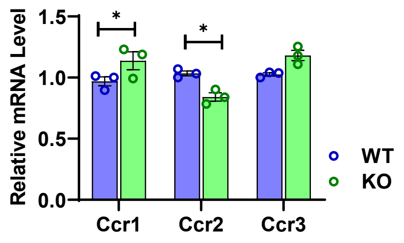

C

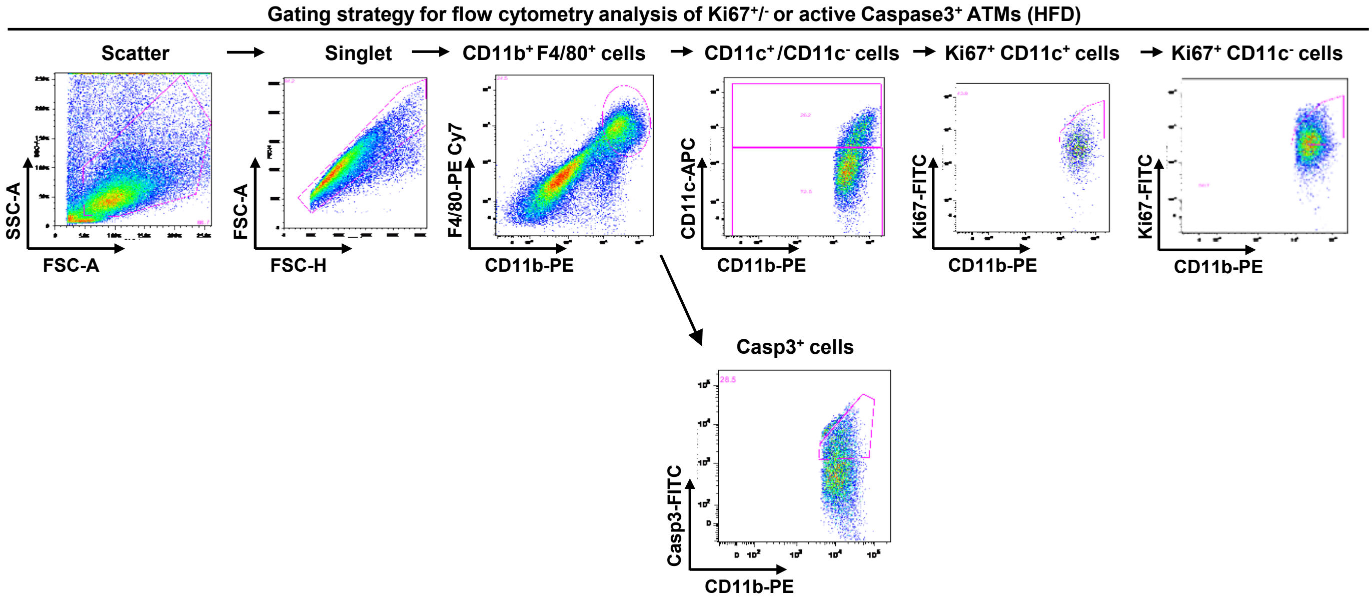

Supplementary Figure 5

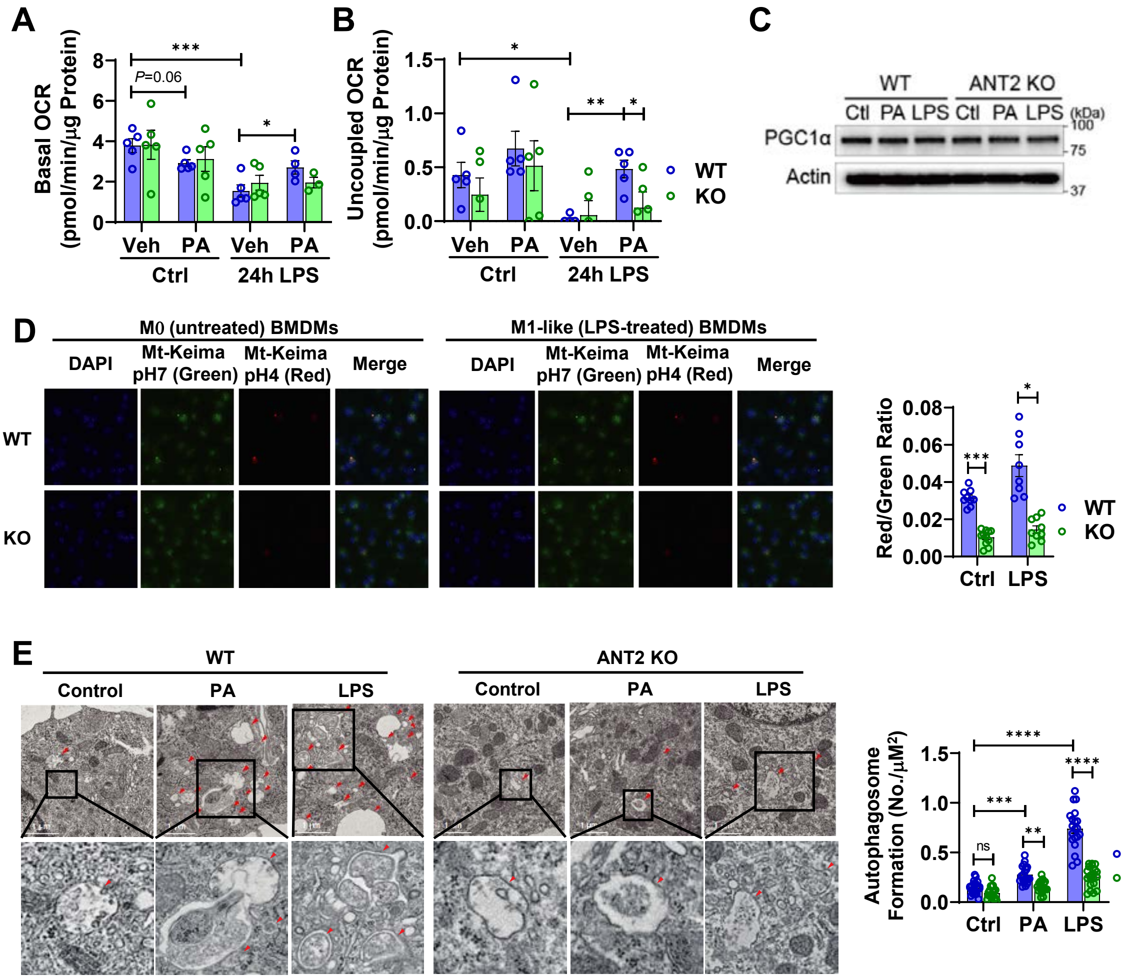

Supplementary Figure 6

A

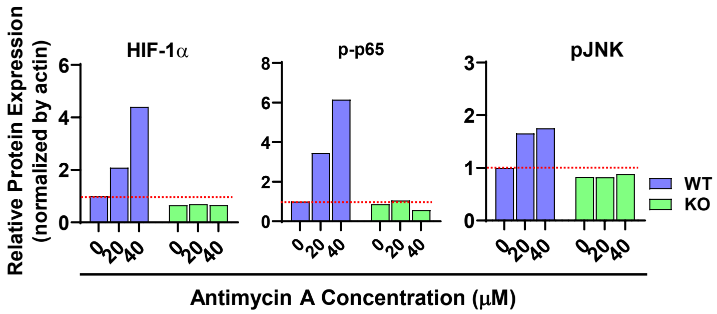

Supplementary Figure 7

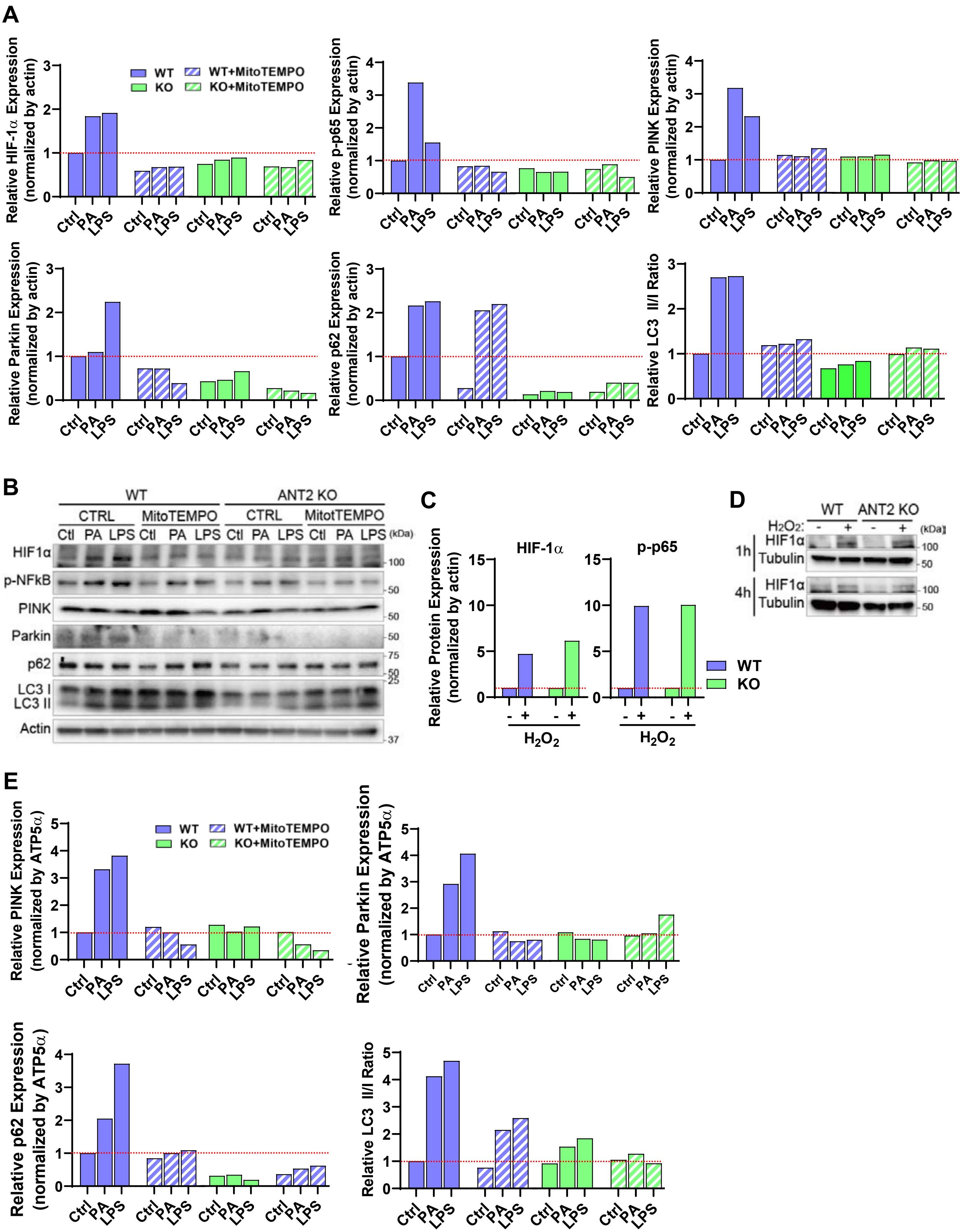

Supplementary Figure 8

Supplementary Table 1. Fast gene set enrichment analysis in CD11c+ ATMs isolated from HFD ANT2 MKO and WT ATMs

Pathway	NES*	Adj *P* value**
HALLMARK_EPITHELIAL_MESENCHYMAL_TRANSITION	3.12	8.9e−29
HALLMARK_OXIDATIVE_PHOSPHORYLATION	2.48	3.8e−14
HALLMARK_ADIPOGENESIS	2.38	2.4e−12
HALLMARK_HYPOXIA	2.21	2.3e−09
HALLMARK_XENOBIOTIC_METABOLISM	2.19	1.3e−08
HALLMARK_GLYCOLYSIS	2.12	4.6e−08
HALLMARK_APICAL_JUNCTION	2.19	5.3e−08
HALLMARK_FATTY_ACID_METABOLISM	2.16	1.1e−07
HALLMARK_CHOLESTEROL_HOMEOSTASIS	2.33	5.2e−07
HALLMARK_PEROXISOME	2.25	6.3e−07
HALLMARK_PI3K_AKT_MTOR_SIGNALING	−0.92	7.0e−01
HALLMARK_SPERMATOGENESIS	−1.04	4.3e−01
HALLMARK_IL6_JAK_STAT3_SIGNALING	−1.20	1.9e−01
HALLMARK_INFLAMMATORY_RESPONSE	−1.45	7.4e−03
HALLMARK_KRAS_SIGNALING_DN	−1.65	2.8e−03
HALLMARK_G2M_CHECKPOINT	−1.57	1.6e−03
HALLMARK_ALLOGRAFT_REJECTION	−1.59	1.3e−03
HALLMARK_E2F_TARGETS	−1.60	5.2e−04
HALLMARK_INTERFERON_ALPHA_RESPONSE	−2.34	5.7e−08
HALLMARK_INTERFERON_GAMMA_RESPONSE	−2.26	1.7e−10

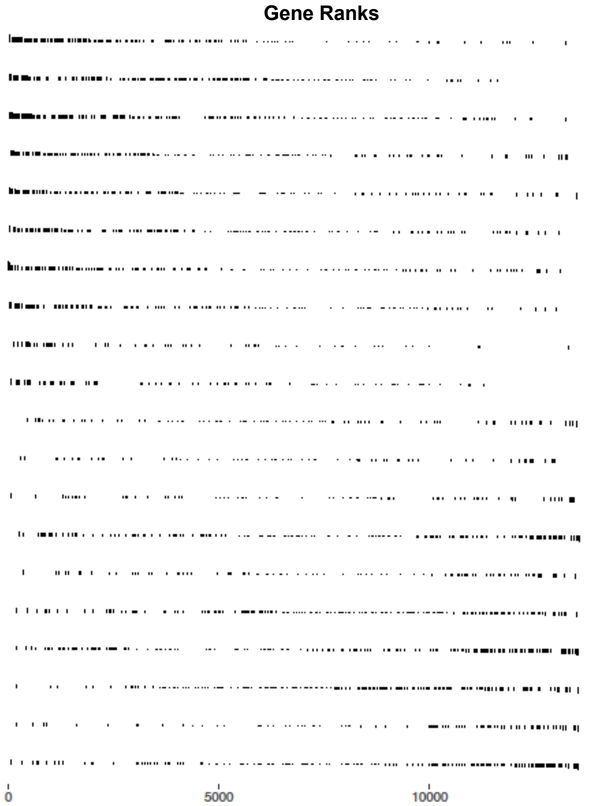

RNA-seq analysis was performed in CD11c⁺ ATMs isolated from ANT2 MKO and WT control mice fed HFD, and the resulting data were subjected to fast gene set enrichment analysis. Positive NES values indicate that the majority of genes implicated in the corresponding pathway or gene network generally showed increased expression in HFD ANT2 KO ATMs compared with HFD WT ATMs, whereas negative NES values indicate that the majority of genes implicated in the corresponding pathway or gene network generally showed decreased expression in HFD ANT2 KO ATMs compared with HFD WT ATMs. The gene rank plot shows fold changes in the expression of each of the genes that are implicated in the corresponding pathway or gene network. Thus, fold changes of the most highly increased genes are plotted on the left, whereas, fold changes of the most highly decreased genes are plotted on the right.
.*NES, normalized enrichment score. ** Adj *P* value, BH adjusted *P* value.

Supplementary Table 2. Detailed Information of The Reagents And Resources

REAGENT or RESOURCE	SOURCE	IDENTIFIER
Antibodies		
Anti-HIF-1α	Abcam	Cat# ab2185; RRID: AB_302883
Anti-ANT1	Abcam	Cat# ab102032; RRID: AB_10710263
Anti-ANT2	Cell signaling	Cat# 14671S; RRID:AB_2798562
Anti-NFκB p65, phospho (Ser536)	Cell signaling	Cat# 3033S; RRID:AB_331284
Anti-SAPK/JNK	Cell signaling	Cat# 9252S; RRID:AB_2250373
Anti-phospho SAPK/JNK, (Thr183/Tyr185)	Cell signaling	Cat# 9255S; RRID:AB_2307321
Anti-SQSTM1/p62 (D6M5X)	Cell signaling	Cat# 23214S; RRID:AB_2798858
Anti-LC3	Cell signaling	Cat# 4108S; RRID:AB_2137703
Anti-Parkin	Abcam	Cat# ab77924; RRID: AB_1566559
Anti-Parkin	Abcam	Cat# ab15494; RRID:AB_301903
Anti-PINK1	Abcam	Cat# ab23707; RRID:AB_447627
Anti-Mitofusin 2	Abcam	Cat# ab56889; RRID:AB_2142629
Total OXPHOS Rodent Antibody Cocktail	Abcam	Cat# ab110413; RRID:AB_2629281
Anti-Akt	Cell signaling	Cat# 4685; RRID: AB_2225340
Anti-phospho Akt (S473)	Cell signaling	Cat# 4060; RRID: AB_2315049
Anti-β-Actin	Sigma	Cat# A2228; RRID:AB_476697
Anti-α-Tubulin	Abcam	Cat# ab4074; RRID:AB_2288001
Anti-CD45 Brilliant Violet 605	BioLegend	Cat# 103139, RRID: AB_2562341
Anti-CD11b PerCP-Cyanine5.5	Thermo Fisher	Cat# 45-0112-82, RRID: AB_953558
Anti-CD11b APC-Cyanine7	BioLegend	Cat# 101226, RRID: AB_830642
Anti-CD11b PE	Thermo Fisher	Cat# 12-0112-82, RRID: AB_2734869
Anti-F4/80 PE-Cyanine7	Thermo Fisher	Cat# 25-4801-82, RRID: AB_469653
Anti-F4/80 PE	BioLegend	Cat# 123110, RRID: AB_893486
Anti-CD11c APC	Thermo Fisher	Cat# 17-0114-82, RRID: AB_469346
Anti-Ki67 FITC	Thermo Fisher	Cat# 11-5698-82, RRID: AB_11151330
Anti-Ki67 PE	Thermo Fisher	Cat# 12-5698-82, RRID: AB_11150954
Anti-FoxP3 PE	Thermo Fisher	Cat# 12-5773-80, RRID: AB_465935
Anti-CD170 (Siglec-F) Brilliant Violet 421	BioLegend	Cat# 155509, RRID: AB_2810421
Anti-CD3 PerCP-Cyanine5.5	BioLegend	Cat# 100218, RRID: AB_1595492
Anti-CD4 APC-Cyanine7	BioLegend	Cat# 100525, RRID: AB_312726
Anti-CD8 Alexa Fluor 488	BioLegend	Cat# 100723, RRID: AB_389304
Anti-IL-4 Brilliant Violet 421	BioLegend	Cat# 504120, RRID: AB_2562102
Anti-IL-17A PE-Cyanine7	Thermo Fisher	Cat# 25-7177-80, RRID: AB_10717952
Anti-IFNγ APC	Thermo Fisher	Cat# 17-7311-82, RRID: AB_469504
Anti-Ly6C PE	Thermo Fisher	Cat# 560592, RRID: AB_1727556
Anti-Ly6G PerCP-Cyanine5.5	BioLegend	Cat# 127616, RRID: AB_1877271
Anti-CD16/CD32	Thermo Fisher	Cat# 14-0161-86, RRID: AB_467135
Rabbit IgG	Sigma	Cat# GLNA 934V
Mouse IgG	Jackson Immuno Research	Cat# 115-035-003, RRID: AB_10015289
Chemicals, Peptides, and Recombinant Proteins		
Palimitic acid	Sigma	Cat# P9767

LPS from *E.coli* O127:B8	Sigma	Cat# L3129
Insulin (Norvolin R)	Norvo Nordisk	Cat# 0169-1833-11
50% Dextrose	Hospira	Cat# 0409-6648-02
IL-4	PeproTech	Cat# 214-14
IL-13	PeproTech	Cat# 210-13
MitoTEMPO	Cayman	Cat# 16621
Collagenase from *Clostridium histolyticum*	Sigma	Cat# C1764
60% high-fat diet	Research Diets	Cat# D12492
Trizol™ Reagent	ThermoFisher	Cat# 15596026
SMARTScribe Reverse Transcriptase	Clontech	Cat# 639537
RBC lysis buffer	eBioscience	Cat# 00-4333-57
Lipofectamine 2000	ThermoFisher	Cat# 11668030
Lipofectamine RNA iMAX	ThermoFisher	Cat# 13778150
Recombinant Mouse M-CSF	Biolegend	Cat# 576406
TransIT®-LT1 Transfection Reagent	Mirus	Cat# MIR2300
Endotoxin-low Bivine Serum Albumin	Sigma	Cat# A8806
LIVE/DEAD™ Fixable Cell Stain Kit	ThermoFisher	Cat# L34966
PKH67 Fluorescent Cell Linker Kits	Sigma	Cat# MINI67-1KT
ROS Detection Reagent	ThermoFisher	Cat# C6827
MitoSOX Red mitochondrial superoxide indicator for live-cell imaging	ThermoFisher	Cat# M36008
Mitochondria Isolation Kit for Cultured cells	ThermoFisher	Cat# 89874
UK5099	Sigma	Cat# PZ0160
BPTES	Sigma	Cat# SML2472
Etomoxir	Sigma	Cat# E1905
Oligomycin	Sigma	Cat# 75351
DNP	Sigma	Cat# 34334
Myxothiazol	Sigma	Cat# T5580
Cyclosporine A	TOCRIS	Cat# 59865-13-3
TRO 19622	TOCRIS	Cat# 22033-87-0
Critical Commercial Assays		
Citrate Synthase Activity Colorimetric Assay Kit	BioVision	Cat# K318-100
Caspase-Glo® 3/7 Assay System	Promega	Cat# G8092
RNeasy Micro Kit	Zymo research	
Insulin ELISA kit	ALPCO	Cat# 80-INSHU-E01.1
MitoProbe™ Transition Pore Assay Kit	ThermoFisher	Cat# M34153
MitoProbe™ JC-1 Assay Kit for Flow Cytometry	ThermoFisher	Cat# M34152
Experimental Models: Cell Lines		
3T3-L1 cell line	ATCC	ATCC® CL-173™
Recombinant DNA		
mKeima-Red-Mito-7	Addgene	Cat# 56018 ; http://n2t.net/addgene:56018 ; RRID:Addgene_56018

CA-HIF1α	Addgene	Cat # 44028 ; http://n2t.net/addgene:44028 ; RRID:Addgene_44028
Software and Algorithms		
GraphPad Prism v.8	GraphPad Software	GraphPad Prism, RRID: SCR_002798
Seahorse Wave v.2.2.0	Agilent	Seahorse Wave, RRID: SCR_014526
FlowJo	FlowJo	FlowJo, RRID: SCR_008520
ImageJ	NIH	https://imagej.nih.gov/ij/

Supplementary Table 3. PCR Primer Sequences

Gene Symbol and Full Name		Forward primer, 5' - 3'	Reverse primer, 5' - 3'
Hif1a	Hypoxia-inducible factor 1-alpha	CAAGATCTCGGCGAAGCAA	GGTGAGCCTCATAACAGAAGCTTT
Hif2a	Hypoxia-inducible factor 2-alpha	TAAAGCGGCAGCTGGAGTAT	ACTGGGAGGCATAGCACTGT
Glut4	Glucose transporter 4	ATGAGAAACGGAAGTTGGAGAG	GTGGGTGCGGCTGCC
Adipoq	AdipoQ, also known as Adiponectin and Acrp30	TGTTCCTCTTAATCCTGCCCA	CCAACCTGCACAAGTTCCCTT
Tnf	Tumour necrosis factor alpha	GAAATGCCACCTTTTGACAGTG	TGGATGCTCTCATCAGGACAG
Ccl5	Regulated on activation, normal T cell expressed and secreted	TGCCCTCACCATCATCCTCAC	GGCGGTTCCTTCGAGTGACA
Ifng	Interferon gamma	ACAGCAAGGCGAAAAAGGATG	TGGTGGACCACTCGGATGA
Ant1	Adenine nucleotide translocase 1	GAATGCTCCCAGATCCCAAGAAT	AAAGGATAGGAAGTCAGGCCA
Ant2	Adenine nucleotide translocase 2	AGAGTGTGACAGCCGTTGC	ACCTTTGAAGAAAGCGTTGGC
Il6	Interleukin 6	GCTACCAAACTGGATATAATC	CCAGGTAGCTATGGTACTCCA
Tgfb	Transforming growth factor beta	GTGGAAATCAACGGGATCAG	ACTTCCAACCCAGGTCCTTC
Itgax	Integrin alpha-X protein	CTGGATAGCCTTTCTTCTGCT	GCACACTGTGTCCGAACTC
Nos2	Inducible nitric oxide synthase	CTCAGCCCAACAATACAAGAT	TGTGGTGAAGAGTGTCATGCA
Arg1	Arginase 1	CTCCAAGCCAAAGTCCTTAGA	AGGAGCTGTCATTAGGGACAT
Il10	Interleukin 10	CCAAGCCTTATCGGAAATGA	TTTTCACAGGGGAGAAATCG
36B4	60S Acidic Ribosomal Protein P0	AGATGCAGCAGATCCGCAT	GTTCTTGCCCATCAGCACC
**Primers used for mouse genotyping**			
Cre	Cre recombinase	TGCAAGTTGAATAACCGGAAA	CTAGAGCCTGTTTTGCACGTT
Primer set 1		ACTCAACCTAGGGCCTTGTG	GGGAGCATTCCTGAAAAATAA
Primer set 2		ACTCAACCTAGGGCCTTGTG	GACTTACCCTCCACGACAGC
**Primers used for mtDNA content assays**			
mtDNA	Mitochondrial DNA	CCCAGCTACTACCATCATTCAAGT	GATGGTTTGGGAGATTGGTTGATGT
18S	18S ribosomal RNA	AGTCCCTGCCCTTTGTACACA	GATCCGAGGGCCTCACTAAAC

Full unedited gel for Figure. 1I

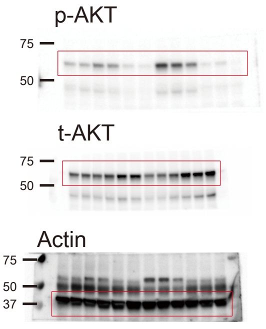

Full unedited gel for Figure. 1J

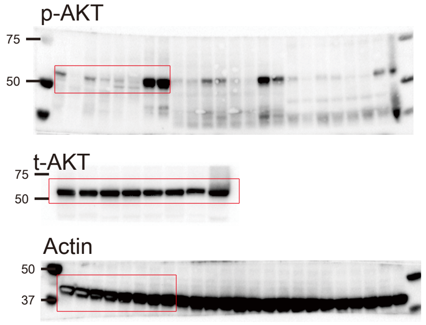

Full unedited gel for Figure. 1K

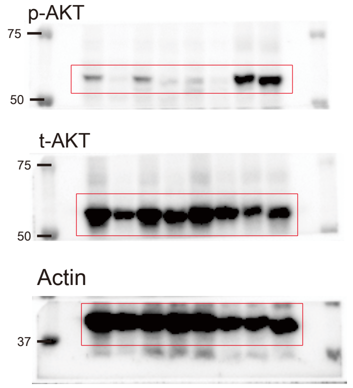

Full unedited gel for Figure. 3G

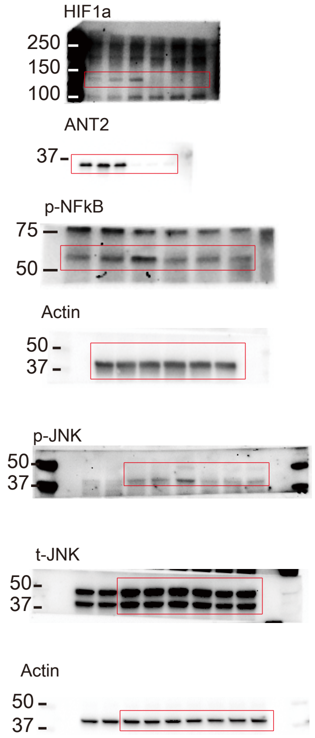

Full unedited gel for Figure. 3J

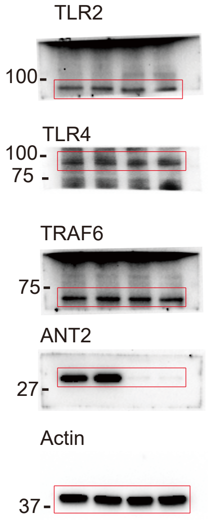

Full unedited gel for Figure. 6D

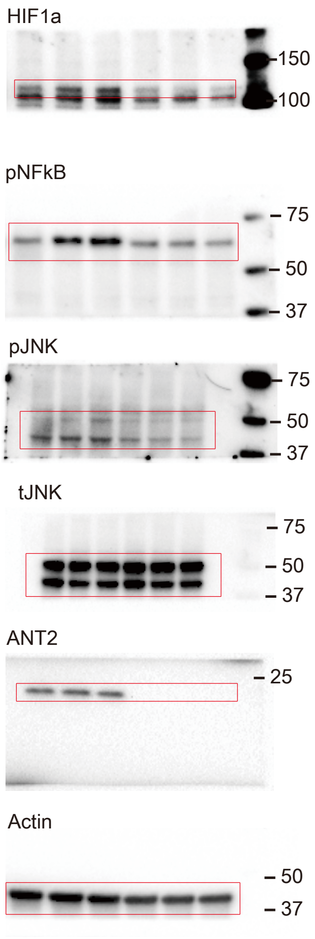

Full unedited gel for Figure. 7A

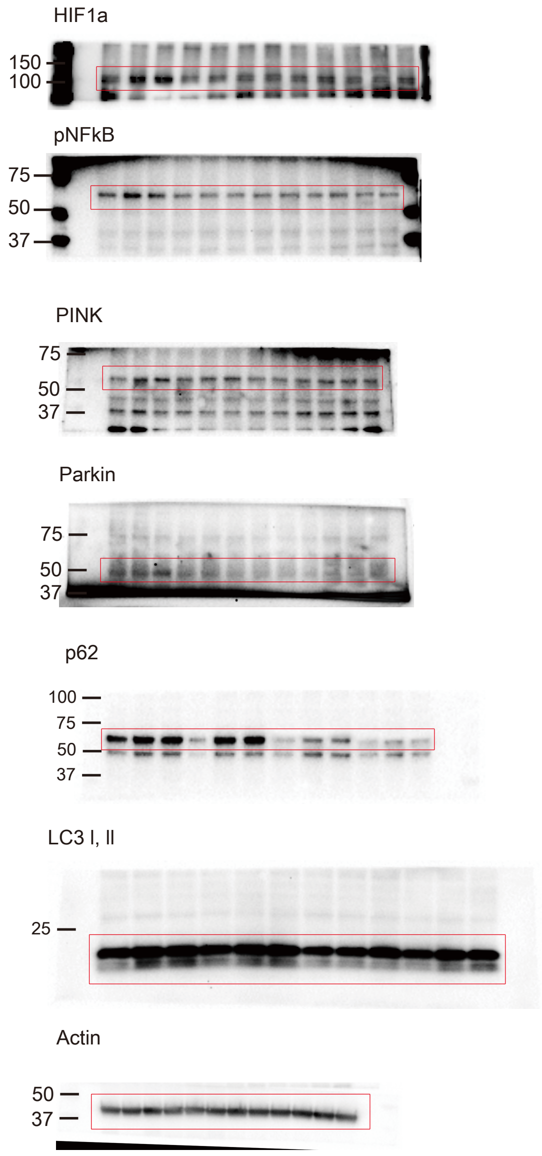

Full unedited gel for Figure. 7C

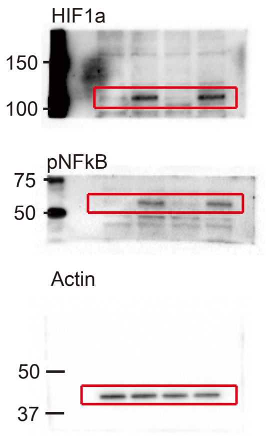

Full unedited gel for Figure. 7F

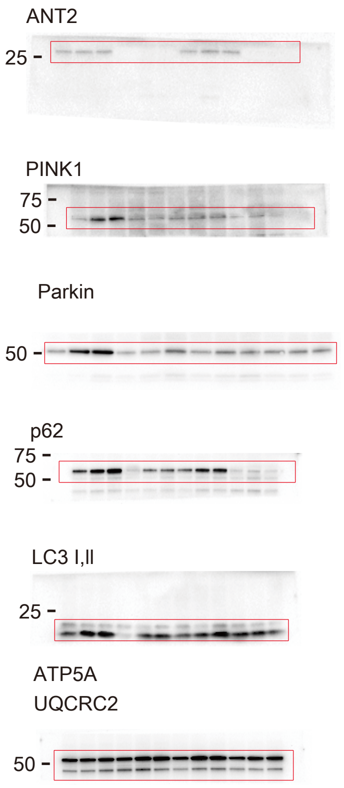

Full unedited gel for Supplementary Figure. 2A

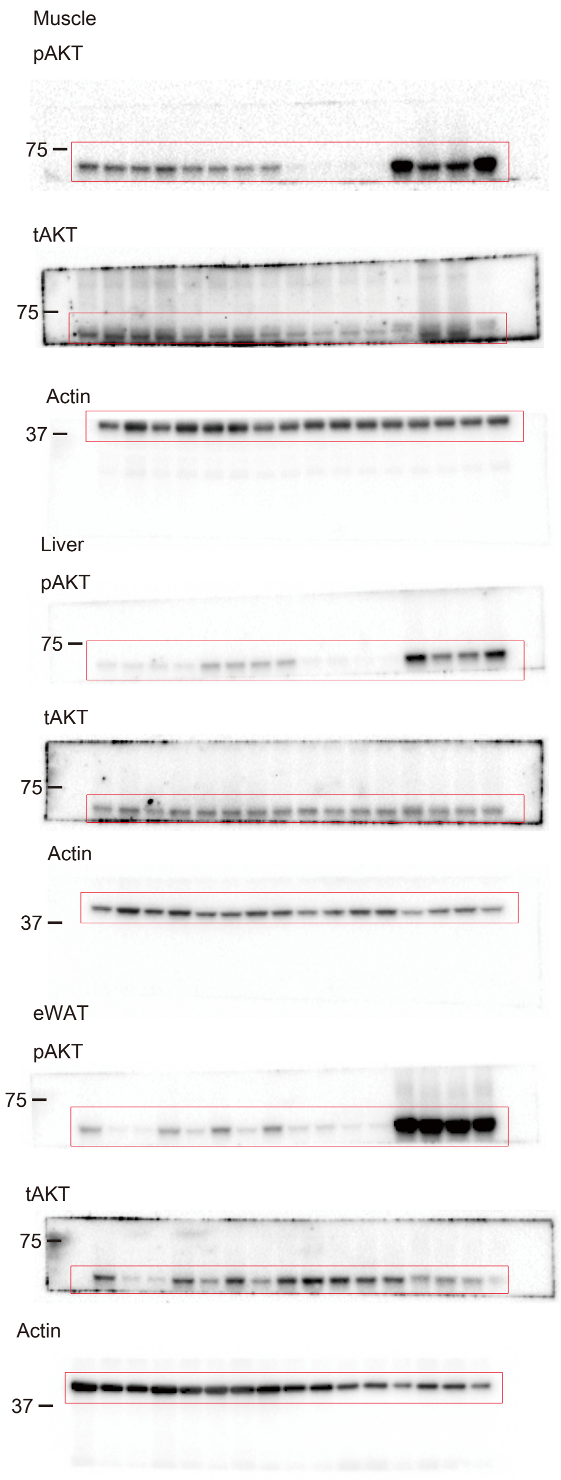

Full unedited gel for Supplementary Figure. 4D

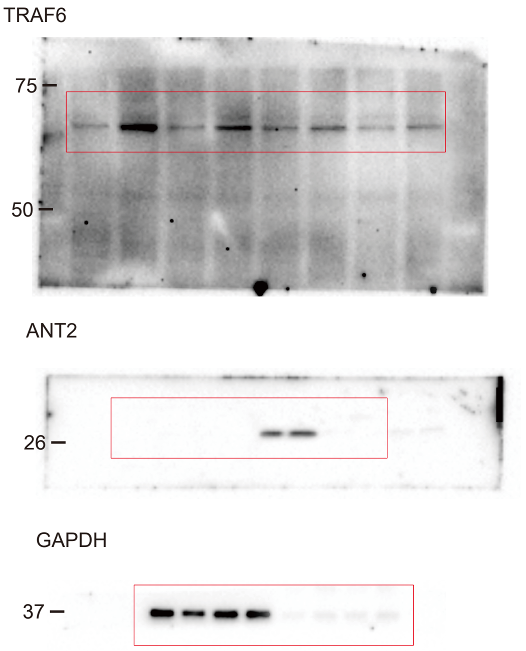

Full unedited gel for Supplementary Figure. 6C

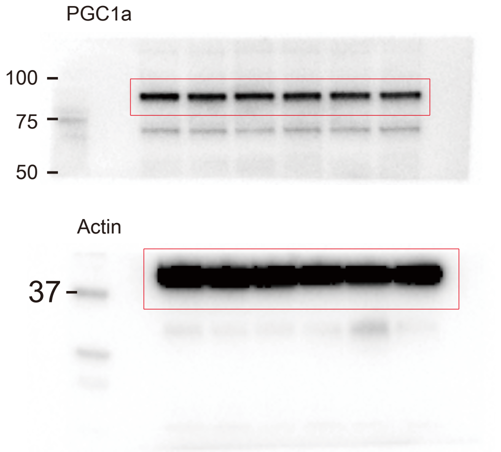

Full unedited gel for Supplementary Figure. 8B

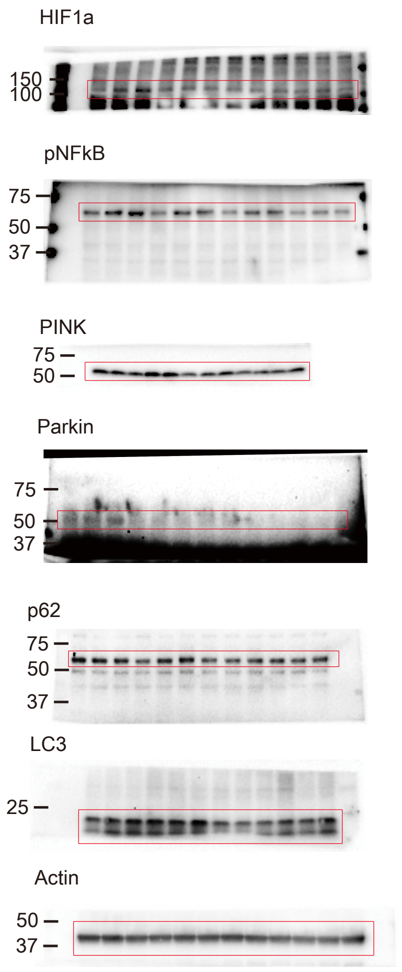

Full unedited gel for Supplementary Figure. 8D

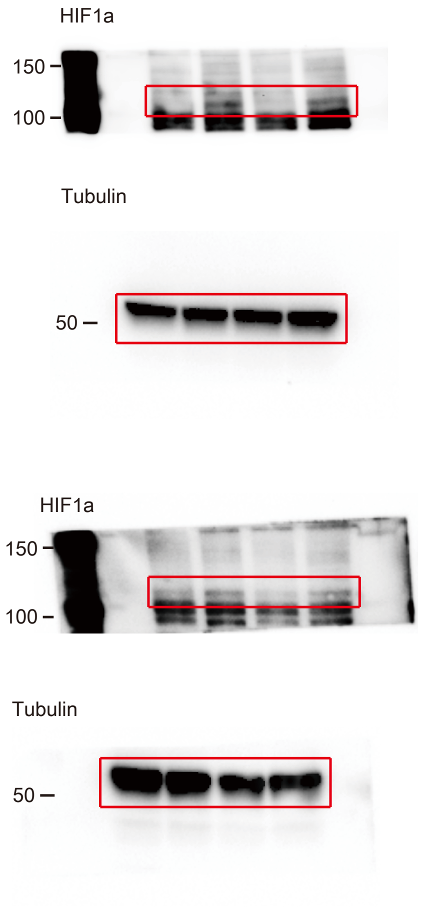

Supplement: Supplemental data [file jciinsight-6-147033-s039.pdf]
